# Macrophages form functional vascular mimicry channels *in vivo*

**DOI:** 10.1038/srep36659

**Published:** 2016-11-11

**Authors:** Faith H. Barnett, Mauricio Rosenfeld, Malcolm Wood, William B. Kiosses, Yoshihiko Usui, Valentina Marchetti, Edith Aguilar, Martin Friedlander

**Affiliations:** 1 Department of Cell and Molecular Biology, The Scripps Research Institute, 10550 N. Torrey Pines Rd, La Jolla, CA 92037, USA

## Abstract

Macrophages, key cells of the innate immune system, are known to support angiogenesis but are not believed to directly form vessel walls. Here we show that macrophages structurally form primitive, NON-ENDOTHELIAL “vessels” or vascular mimicry (VM) channels in both tumor and angiogenesis *in vivo* models. These channels are functionally connected to the systemic vasculature as they are perfused by intravenously injected dye. Since both models share hypoxic micro-environments, we hypothesized that hypoxia may be an important mediator of VM formation. Indeed, conditional genetic depletion of myeloid-specific HIF-1α results in decreased VM network formation, dye perfusion and tumor size. Although the macrophage VM network shares some features with an endothelial vasculature, it is ultrastructurally different. Cancer stem cells have been shown to form vascular mimicry channels. Our data demonstrates that tumor-associated macrophages also form them. The identification of this novel type of vascular mimicry may help in the development of targeted cancer therapeutics.

Angiogenesis, the growth of new blood vessels from pre-existing ones, is required for wound healing, menstruation, embryogenesis and is also critical in various pathological conditions including tumor growth. Angiogenesis is induced by growth factors and/or by low oxygen conditions through hypoxia-inducible factors (HIFs)^1^. In this process, dormant endothelial cells lining the lumens of pre-existing blood vessels are stimulated to proliferate, remodel the extracellular matrix (ECM), migrate and differentiate to form the walls of newly created vessels in response to angiogenic cues^2^.

Although endothelial cells (EC) line mature blood vessels, there are examples of non-endothelial cells forming vascular channels. In the ischemic heart, macrophages “drill” channels that are not lined by endothelial cells and serve as an alternative microcirculation^3^. Another example is the vessel lumens of invertebrates which are created by phagocytes, the ancestral relative of the macrophage^4^,^5^. A third example is found in a variety of solid tumors as their vasculature is comprised of both endothelial-lined blood vessels and a non-endothelial microcirculation. The latter is called vascular mimicry (VM)^6^ and is believed to occur through the differentiation of cancer stem cells into endothelial-like cells^7^,^8^,^9^,^10^.

In physiologic and developmental angiogenesis, macrophages are thought to play a supportive role^11^ as they promote blood vessel outgrowth through cytokine secretion and remodeling of the ECM^12^,^13^. They also serve as bridging cells enabling anastomoses of neighboring endothelial tip cells^14^ and as promoters of retinal vasculature remodeling^11^. Additionally, although still controversial, myeloid precursor cells and macrophages have been shown to differentiate into endothelial-like cells both *in vitro* and *in vivo*^13^,^15^,^16^ and/or mural cells^17^,^18^. However, a role for macrophages in the direct formation of a functional conduit system distinct from the endothelial vasculature has not been described.

Furthermore, macrophages are also involved in pathological angiogenesis. Tumor-associated macrophages can make up to 50% or more of the cells within a broad spectrum of solid tumors including breast cancer, glioblastoma, melanoma and lung cancer, among others^19^. Tumors recruit macrophages and are believed to reprogram them to serve as the major source of angiogenic factors^20^,^21^. As in physiological and developmental angiogenesis, macrophages in tumors are believed to play a supportive role in this process.

Here we show that macrophages have a new and unrecognized structural role in the formation of a primitive, perfused vascular mimicry network in both *in vivo* angiogenesis and tumor models. This network shares some features with those composed of arterial, venous and lymphatic endothelium. However, it is ultrastructurally different and can be misidentified as an endothelial vasculature because the macrophages that form it also express endothelial markers. The structural involvement of macrophages in hypoxia-driven vascular mimicry may provide additional targets for therapeutic intervention in cancer and other vascular diseases.

## Results

### Macrophages form a network of interconnected cells

**Figure 1:**
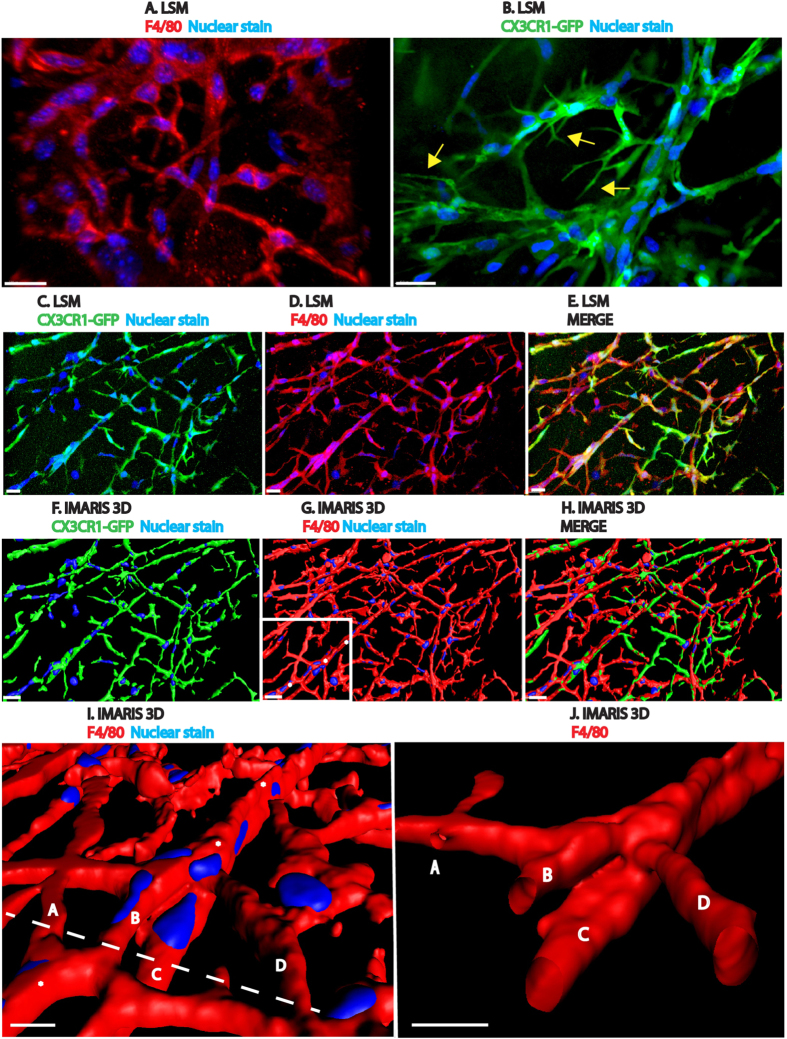
Monocytes/macrophages form a tubular network in both wild type and CX3CR1^*GFP/GFP*^ mice. (**A–J**) All images are generated from confocal Z stacks. (**A**) Immunohistochemistry for F4/80 was performed on plugs isolated from wild type C57BL/6J mice 10 days after matrigel injection. F4/80^+^ cells interconnect to form a tubular network. F4/80 is shown in red and Hoechst nuclear stain in blue. (**B**) CX3CR1^*GFP/GFP*^ cells at the network’s leading front, shown in green, are extending filopodia (yellow arrows) in a 10 day plug. (**C–E**) Images of individual and merged channels show a 3-dimensional network of CX3CR1^*GFP/GFP*^ cells (green) that are also F4/80^+^ (red). Nuclear stain in blue. (**F–H**) Imaris 3D rendering of (C–E) images. In this image, there are 204 cells of which 82% are F4/80^+^, 76% are CX3CR1^*GFP/GFP*^ lineage and 54% are both CX3CR1^*GFP/GFP*^ and F4/80^+^. (**I**) Magnified view of a multinucleated tube (asterisks) within boxed area in (**G**). Letters A–D represents different branches within the structure. (**J**) Transverse view along dashed line in (I) demonstrates a multi-branched F4/80^+^ tubular structure and their corresponding lumens (nuclei removed). Scale bars are 20 um. N = 3–4 per group.

The subcutaneous matrigel angiogenesis assay was utilized to study cellular migration and the intercellular interactions of monocytes/macrophages (MACs) and endothelial cells *in vivo*. Interferon-supplemented liquid matrigel was initially injected subcutaneously into the flank of 6–8 week-old wild type C57BL/6J mice. Plugs were explanted 10 days after injection, cleaned of any associated tissues, fixed, sectioned and immunostained with various antibodies including the macrophage marker, F4/80, and the endothelial marker, CD31. Surprisingly, fluorescent confocal microscopy analysis revealed that a complex and interconnected cellular network existed in the plug’s interior assembled by F4/80^+^ cells (Fig. 1A), with a subset of them also expressing CD31 (SI. 1A,B).

To determine if cytokine supplementation was critical to network formation, pilot experiments were performed with matrigel supplemented with either pro-angiogenic (VEGF) or pro-inflammatory cytokines (Interferon and/or GM-CSF). Both VEGF and the inflammatory cytokines resulted in a qualitatively and morphologically similar macrophage network (SI. 2A–H). These results showed that the macrophage network formed with either pro-inflammatory or angiogenic cytokines and further matrigel experiments were performed with interferon supplementation.

To further determine if this network was comprised by MACs, the experiment was repeated in CX3CR1^*GFP/GFP*^ and CX3CR1^*GFP/*+^ mice. These mice allow lineage tracing of cells of monocyte, macrophage, natural killer and microglial origin by detecting their green fluorescent protein expression when examined by confocal microscopy. These mice are fertile and have no overt tissue or behavioral abnormalities (Jackson Laboratory, Bar Harbor, Maine).

Our experiments showed that the macrophage network seen in subcutaneous plugs of wild type mice was also present in plugs explanted form CX3CR1^*GFP/GFP*^and CX3CR1^*GFP/*+^ mice (Fig. 1B). Here, GFP^+^ cells formed a network of interconnected cells and many of its leading fronts exhibited polarized CX3CR1^GFP+^ cells with filopodial extensions (arrows Fig. 1B). To further clarify whether the network was formed of myeloid and not endothelial cells, we immunostained for the macrophage marker F4/80 and demonstrated that the cells forming the GFP^+^ lattice-like network also expressed F4/80 (Fig. 1C–E). This result indicated that the network was formed of myeloid and not endothelial cells.

To evaluate the cell to cell interactions in greater detail, Imaris 3D renderings were performed on the imported confocal Z-stack images depicted in Fig. 1(C–E). For a detailed explanation of this software, refer to Materials and Methods. Briefly, the software collects an intensity value per color channel and provides a way to examine cell to cell interactions in 3 dimensions. Furthermore, Imaris enables cross sectional analysis of 3D biological structures.

The Imaris 3D renderings (Fig. 1F–H) proved to be faithful representations of the LSM confocal Z-stacks (Fig. 1C–E). The multinucleated tubular network is multi-branched, consisting of tubes extending 200 um or more and composed of F4/80^+^, CX3CR1^GFP+^ macrophages (Fig. 1I). A transverse cut through the multinucleated structure (Fig. 1I) and its branches confirmed its tubular nature (Fig. 1J, SI. 1C, SI. movie1).

### Macrophages invade plug, form network and may express CD31 prior to appearance of blood vessels

**Figure 2:**
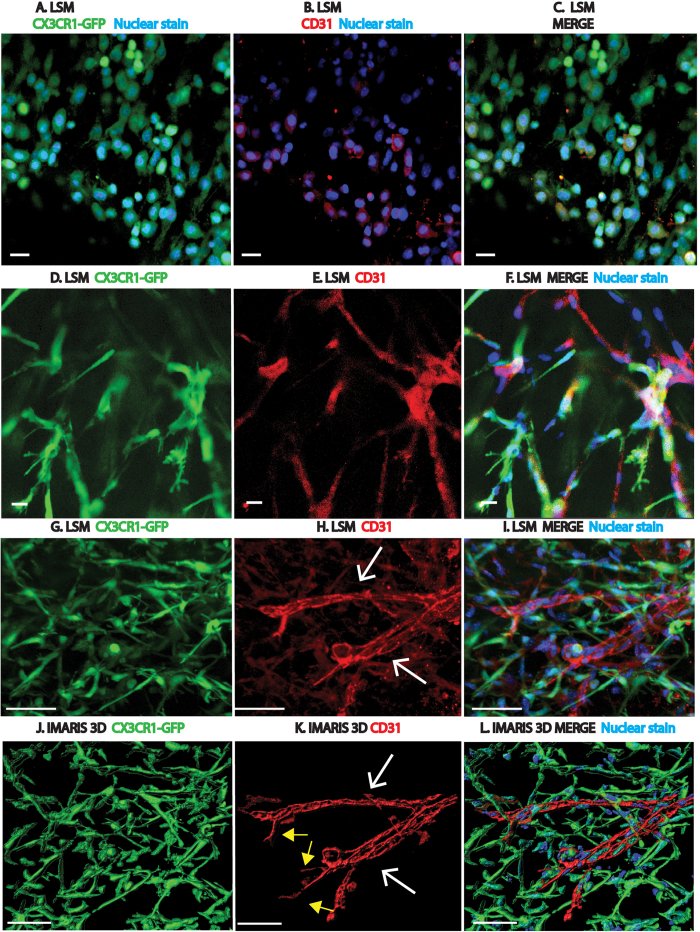
Time course of macrophage network formation. (**A–L**) Time course of network formation in plugs isolated from CX3CR1^*GFP/GFP*^ mice. CX3CR1^*GFP/GFP*^ cells shown in green, CD31 in red, and Hoechst nuclear stain in blue. All images are Z stacks with representative images shown at 2 hours, 5 and 10 day time points. (**A–C**) Images show invading cells within the plug 2 hours after matrigel injection. 131 invading cells were counted, of which 33% were single positive for CX3CR1^*GFP/GFP*^, 18% were single positive for CD31 and 41% were double positive for both markers (**D–F)** Invading CX3CR1^*GFP/GFP*^ cells elongate and form cord-like structures by 5 days. Quantitative analysis revealed that 67% were CX3CR1^*GFP/GFP*^, 7% CD31 and 8% double positive. (**G–I**) A complex CX3CR1^*GFP/GFP*^tubular network is formed by 10 days following matrigel injection. Quantitative analysis revealed that 64% were CX3CR1^*GFP/GFP*^, 36% were CD31^+^ and 30% were double positive. A few structures resembling traditional endothelial blood vessels (white arrows) with apparent tip cell filopodia (yellow arrows) are seen. These endothelial blood vessel-like structures are CX3CR1^*GFP/GFP*^ negative, CD31 positive. (**J–L**) represent Imaris 3D renderings of (**G–I).** Scale bars are 20 um. N = 4–6 per group. All images are representative images with greater than 265 2D images or Z stacks examined.

To study this process as a function of time, matrigel was subcutaneously injected into CX3CR1^*GFP/GFP*^ mice, explanted at various time points (0.5 hours, 2 hours, 24 hours, 3, 4, 5 and 10 days) and immunostained for various cell surface markers including CD31 (Fig. 2). At the half hour time point, there was evidence of some CX3CR1^*GFP/GFP*^ cellular invasion into the plug and the numbers increased by 2 hours, at which time some of these cells also expressed CD31 (Fig. 2A–C). The myeloid cells in the plug may represent either neighboring macrophages from adjacent tissues, monocytes from the blood extravasating into the plug or a combination of both. Between 24 hours and 5 days following matrigel injection, CX3CR1^*GFP/GFP*^ cells elongated and aligned with adjacent cells to form interconnected cords (Fig. 2D–F) that matured into a more complex 3 dimensional tubular network by day 10 (Fig. 2G–I). Analysis of a plug section representative of a 10 day subcutaneous plug revealed that 64% of the cells were associated with the green signal (CX3CR1^GFP^) and 36% with the red (CD31); the latter cells appearing to form traditional endothelial blood vessel walls by both LSM and 3D Imaris rendering (Fig. 2H,K white arrows). These results showed that macrophages formed a robust multicellular vascular mimicry (VM) network prior to the appearance of endothelial vessels *in vivo*. This experiment was repeated in the CX3CR1^*GFP/*+^ mouse with similar results.

### Macrophages interact with the angiogenic front of the invading endothelial blood vessels

To study the interactions between macrophages and endothelial cells more closely, Imaris software was used to render three-dimensional images (Fig. 2J–L, SI. 3). In order to analyze the angiogenic front, only the CD31 signal in the region of interest was three dimensionally rendered (Fig. 2H,K, SI. 3B,E,H,K). Here, the CD31 signal corresponded to cells that appeared to form blood vessels, while the CX3CR1^*GFP/GFP*^ cells formed a network of interconnected cells that intimately interacted with the growing blood vessel front (SI. 3). Importantly, the two cell types appeared to be distinct.

### Macrophages express extracellular matrix (ECM) degrading proteases

An invading endothelial front is characterized by expression of ECM degrading proteases^22^. Therefore, to determine if the observed macrophage network also expressed proteolytic enzymes, we immunostained sections of subcutaneous plugs for MMP9 and ADAM17. Our results showed that from 2 hours to 10 days after matrigel injection, CX3CR1^*GFP/GFP*^ cells express ADAM17 and MMP9 (SI. 4A–J). Furthermore, RT-qPCR analysis confirmed that F4/80^+^ macrophages isolated from subcutaneous plugs of C57BL/6J mice expressed MMP 2, 9, 14 and 19 (SI. 5).

To examine the relationship between the cells and the matrigel, collagen IV staining was done to identify the matrigel. CX3CR1^*GFP/GFP*^cells localized to channels within matrigel (zones without collagen IV) (SI. 4K). These channels were not present in control matrigel solidified at 37 °C (SI. 6A,B).

### Cells forming the VM network express both macrophage and endothelial markers

Additional immunohistochemical studies were performed for various macrophage and endothelial markers. While most of the cells forming the network expressed various macrophage markers, such as F4/80, CD163 (hemoglobin/haptoglobin scavenger receptor), CD11b, MSR1 (macrophage scavenger receptor-1), and CD206 (mannose receptor), not all of them were CX3CR1^GFP+^ (SI. 7A–M). In addition, CX3CR1^*GFP/GFP*^ cells also expressed the endothelial cell surface markers CD31, CD34, TIE2, and Phospho-Erk (an endothelial tip cell marker)^23^ (Fig. 2, SI. 8A–J).

Additional experiments were performed on subcutaneous plugs explanted from wild type C57BL/6J mice. A network of interconnected cells was identified that expressed the myeloid marker, CD11b and the endothelial lymphatic marker, LYVE1 (SI. 8K–M). This result showed that although there were variations in cell surface marker expression patterns, the principal cells forming the network were macrophages and not ECs.

To validate our immunohistochemistry data and look at a broader survey of markers associated with angiogenesis, we dissociated 3-day plugs, sorted for cells expressing the macrophage marker F4/80, isolated RNA and ran RT-qPCR using an angiogenesis array (SI. 5). The results showed that the F4/80^+^ cells expressed genes for multiple cytokines, HIF-1α and genes generally associated with endothelial cells, including PECAM-1, Endoglin, VE-Cadherin, and neuropilins-1,2.

### Blocking monocyte to macrophage maturation leads to a decrease in VM macrophages

**Figure 3:**
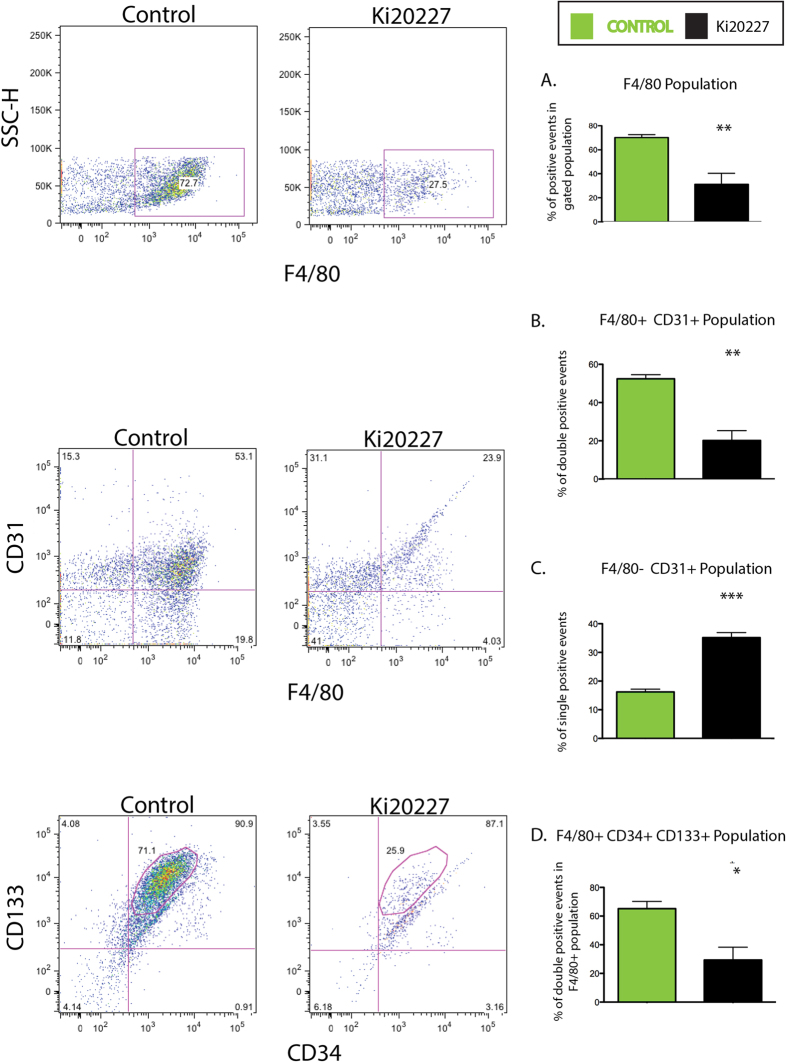
Inhibition of monocyte to macrophage maturation leads to a decrease in VM macrophages. (**A–D**) Mice with sc matrigel plugs were treated with the c-Fms tyrosine kinase inhibitor Ki20227, a drug that blocks monocyte to macrophage maturation. Seven days post matrigel injection, mice were euthanized, plugs harvested, cells isolated from subcutaneous plugs, immunolabeled with antibodies against F4/80, CD31, CD34 and CD133 and subjected to flow cytometry. (**A**) Ki20227 decreases F4/80^+^ macrophages by 67%. (**B**) F4/80^+^, CD31^+^ population decreases by 62%. (**C**) F4/80^**−**^, CD31^+^ population doubled in response to Ki treatment. (**D**) Ki treatment led to a 61% decrease in the F4/80^+^, CD34^+^, CD133^+^ population. This figure represents one experiment with an N = 4–5 mice per group, flow data shown for one plug isolate and significance indicated above each bar graph. Experiment was repeated 2 times with similar results.

To study changes in cell populations within the plug, we treated plug-bearing mice with the monocyte to macrophage maturation inhibitor, Ki20227 (Ki). Systemic treatment with Ki or vehicle was started 2 days prior to subcutaneous matrigel injection and continued daily until plugs were explanted 7 days post implantation. Cells were isolated and stained for flow cytometry with antibodies to the macrophage marker F4/80 as well as antibodies to the endothelial and stem cell markers CD31, CD34^24^ and CD133. CD133 is expressed by hematopoietic stem cells, endothelial progenitor cells (EPC) and cancer stem cells, although the role of CD133 as an EPC marker has been re-evaluated^25^. In Ki-treated mice, the F4/80^+^ cell population decreased by 67% (Fig. 3A). Similarly, the F4/80^+^, CD31^+^ population decreased by 62%, as did the F4/80^+^, CD34^+^, CD133^+^ (triple positive) macrophage population by 61%; suggesting that the majority of the F4/80^+^ cells also expressed CD31, CD34 and CD133 (Fig. 3B,D). Interestingly, the Ki-related decrease in macrophages correlated with a doubling of the F4/80^−^, CD31^+^ population, a presumed endothelial cell population (Fig. 3C). Furthermore, similar results were also found with clodronate, a drug that induces apoptosis of macrophages. Clodronate decrease the F4/80^+^, CD45^+^ cell population with a compensatory increase in the endothelial CD34^+^, CD45^−^ population (data not shown). Importantly, the largest population of cells within the plug was the VM macrophage and not endothelial cells.

### By electron microscopy, the tubular structures are not an endothelial vasculature

**Figure 4:**
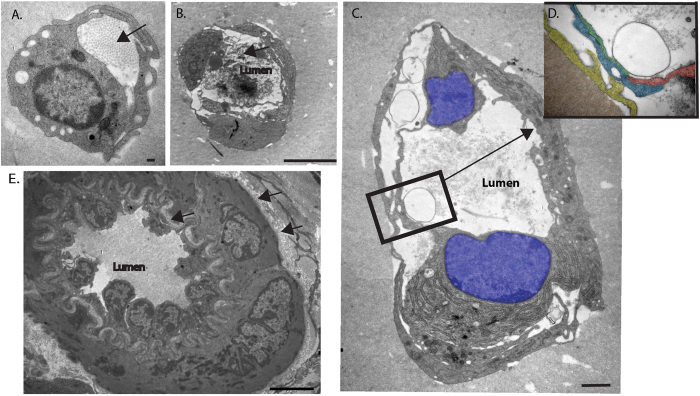
EM analysis of macrophage interactions in sc matrigel. **(A–E**) Electron microscopy (EM) of matrigel showing predominate cell architecture at different time points. (**A**) EM of s.c. plug at 3 days. Single cells enwrap a channel devoid of matrigel. Within this channel, there is a bundle of collagen (arrow). Cell processes overlap. (**B**) EM of s.c. plug at 7 days. Two or more cells line the channel. Collagen (arrow) and debris is seen between two cells in a zone without matrigel. The cells have overlapping cell processes. (**C**) EM of s.c. plug at 11 days. At least two cells (nuclei are colored blue) line the potential lumen. (**D**) magnified view of box in (**C**) to show overlapping cell processes. Each process is colorized to show 4 separate processes.(**E**) For comparison, a blood vessel from plug surface is shown. Collagen is on the abluminal side of blood vessel and the endothelial cell junctions are end-to end. Scale bars represent 5 um.

To further characterize the cellular network formed by macrophages within matrigel, subcutaneous plugs were studied by transmission electron microscopy (TEM) at 3, 7, 11 and 14 days and by scanning EM (SEM) at 11 days. At the 3-day time point, predominately single cells were identified within cleared zones of matrigel (Fig. 4A) and collagen bundles were encircled by cell processes (Fig. 4A arrow). At later time points, the cells interacted to form potential tubular structures. A cross section through two potential tubular structures are shown (Fig. 4B,C). In these typical examples, two cell bodies are juxtaposed at the margins of zones of matrigel clearance (Fig. 4B,C). In certain areas, collagen is identified between the two cells (Fig. 4B arrow). The vessel-like structures within the matrigel had several features that distinguished them from endothelial blood vessels: (1) collagen was located within the central cleared zone, or potential lumen (Fig. 4B), rather than on the abluminal surface as seen with traditional blood vessels (Fig. 4E black arrows); and (2) cells exhibited numerous overlapping cell processes (Fig. 4D) rather than the end-to-end cell junctions characteristic of endothelial cells in blood vessels. An additional feature was swollen rough endoplasmic reticulum consistent with active protein synthesis (SI. 6F). In over 400 images, no classic blood vessels were identified within the substance of the matrigel. A traditional blood vessel from the surface of a matrigel plug is included for comparison (Fig. 4E). These observations in addition to our confocal results suggested that these were probable vascular mimicry channels.

### The VM network is a conduit for systemically injected dye

**Figure 5:**
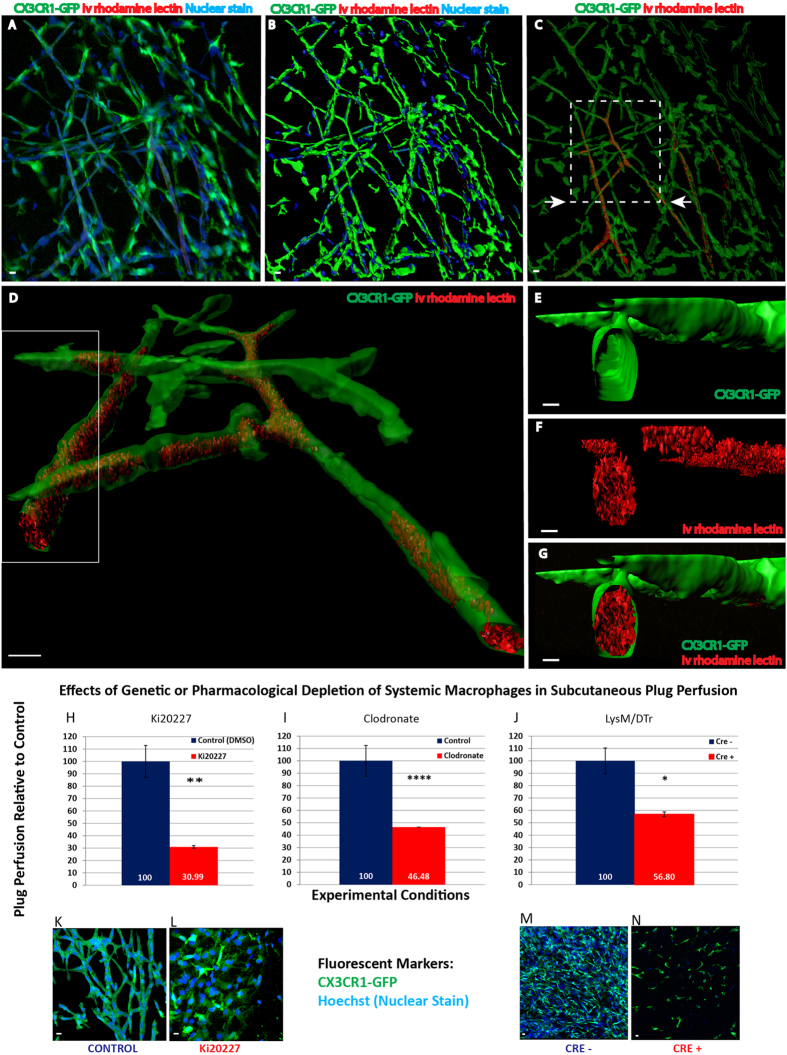
The macrophage network is a functional vasculature. (**A–G**) Various representations of a 32 *μm* Z-stack confocal image from a 9 day matrigel plug removed from a CX3CR1^*GFP/GFP*^ mouse. Mice were i.v. injected with rhodamine lectin. All Scale bars indicate 10 *μm*. (**A**) LSM image of CX3CR1^*GFP/GFP*^ network (green), nuclei (blue) and i.v. rhodamine lectin (red). Rhodamine lectin is within CX3CR1^*GFP/GFP*^tubes and therefore not seen in merged image. Network consisted of 298 CX3CR1-GFP^+^ cells (82% of total cells) covering 26,198 μm^2^. N = 4. (**B**) Imaris 3D rendering of (**A**). (**C**) The green CX3CR1^*GFP/GFP*^ Imaris isosurface is rendered translucent showing rhodamine lectin within the CX3CR1 tubular network. (**D**) Boxed area in image (**C**) is rotated to the left and raised approximately 30 degrees relative to the horizontal axis. A portion of the Z stack is shown. (**E–G**) Transverse cut through one of the branches shows dye within this conduit. (**H–J**) **Perfusion assays**. Mice were injected with matrigel and treated with Ki20227 (**H**) or clodronate (**I**). Subsequently, mice were intravenously injected with 3 KD dye, plugs harvested and dye within the plugs measured by spectrophotometry. Ki20227 treatment reduces perfusion by 70% relative to control. (**I**) Depletion of macrophages with clodronate reduces perfusion by 54% relative to control. (**J**) Conditional genetic depletion of macrophages in LysM-Cre(+); R26^iDTR/+^; CX3CR1^GFP/+^ littermates bearing plugs reduces perfusion by 44% relative to control. Data for perfusion assays represents combined results of 2–3 experiments. (**K–N**) Representative images of perfusion experiments. (**K**) **Ki Control:** 123 cells cover a surface area of 62,078 μm^2^ and a volume of 68,378 μm^3^. **(L) Ki-treatment:** 88 cells cover 75,364 μm^2^ (75,562 μm^3^). **(K–L)** 8.88 μm confocal Z-stacks sections. **(M)** LysM-Cre(−); R26^iDTR/+^; CX3CR1^GFP/+^: 879 cells were detected, 735 were CX3CR1^*GFP*/+^ macrophages covering 381,007 μm^2^ (275,292 μm^3^). **(N)** LysM-Cre(+); R26^iDTR/+^; CX3CR1^GFP/+^: 91 total cells, 76 of which were CX3CR1^*GFP*/+^ macrophages covering 50,741 μm ^2^ (56,893 μm^3^). **(M-N)** Z-stacks were 14.45 μm and 13.6 μm for LysM-Cre(−); R26^iDTR/+^; CX3CR1^GFP/+^ and LysM-Cre(+); R26^iDTR/+^; CX3CR1^GFP/+^, respectively.

To determine if the tubular network was perfused, we intravenously injected rhodamine-labeled GS lectin (114 Daltons) into CX3CR1^*GFP/GFP*^mice bearing 9 day subcutaneous matrigel plugs. Plugs were removed 5 minutes following tail vein injection and processed for confocal microscopy. GS lectin was observed within regions of the CX3CR1^*GFP/GFP*^network (Fig. 5A–G). In this representative image, 25% of the macrophage VM network contained 79% of the dye.

Using confocal and EM imaging, we did not observe red blood cells (RBCs) within the CX3CR1-GFP tubules. However, GS lectin can stain galactosyl residues on macrophages. To rule out the possibility that lectin was leaking into the network and staining the outside of the macrophages rather than passing through the lumen, we repeated the experiment using a 3 KD rhodamine dextran that does not stain macrophages. As with the GS lectin, the 3 KD dextran was found within the VM network supporting that macrophages form a functional, primitive conduit system that connects to the systemic endothelial vasculature.

### Depletion of macrophages decreases plug perfusion

To confirm that macrophages formed a perfused tubular network, we used three approaches to deplete the macrophage population. First, monocyte to macrophage maturation was blocked with intraperitoneal injection of Ki20227 (Ki). Mice bearing subcutaneous matrigel plugs were treated daily with Ki or vehicle and 8 days later intravenously injected with 3 KD fluorescent dextran. Plugs were then explanted and dye within plugs analyzed spectrophotometrically. In Ki-treated mice, there was a 69% decrease in dye within the plugs (Fig. 5H,K,L). Second, liposomal clodronate was used as an alternative method to deplete macrophages. Phagocytosis of liposome-encapsulated clodronate by MACs induces apoptosis and depletion of systemic MACs. Daily intraperitoneal injection of lipo-clodronate compared with liposome alone led to a 54% reduction in perfusion (Fig. 5I). Third, we used LysM-Cre(+); R26^iDTR/+^ transgenic mice to conditionally deplete myeloid cells in adult mice by rendering them sensitive to diphtheria toxin. In this experiment, matrigel was subcutaneously injected into LysM-Cre; R26^iDTR/+^ transgenic mice and Cre^+^ and Cre^−^ littermates were treated with diphtheria toxin. Results indicated that Cre^+^ myeloid-depleted mice had a 43% reduction in subcutaneous matrigel plug perfusion relative to the Cre^−^ control group (Fig. 5J,M,N).

### Macrophage-specific expression of HIF-1α is a driver of network formation and supports tumor growth

**Figure 6:**
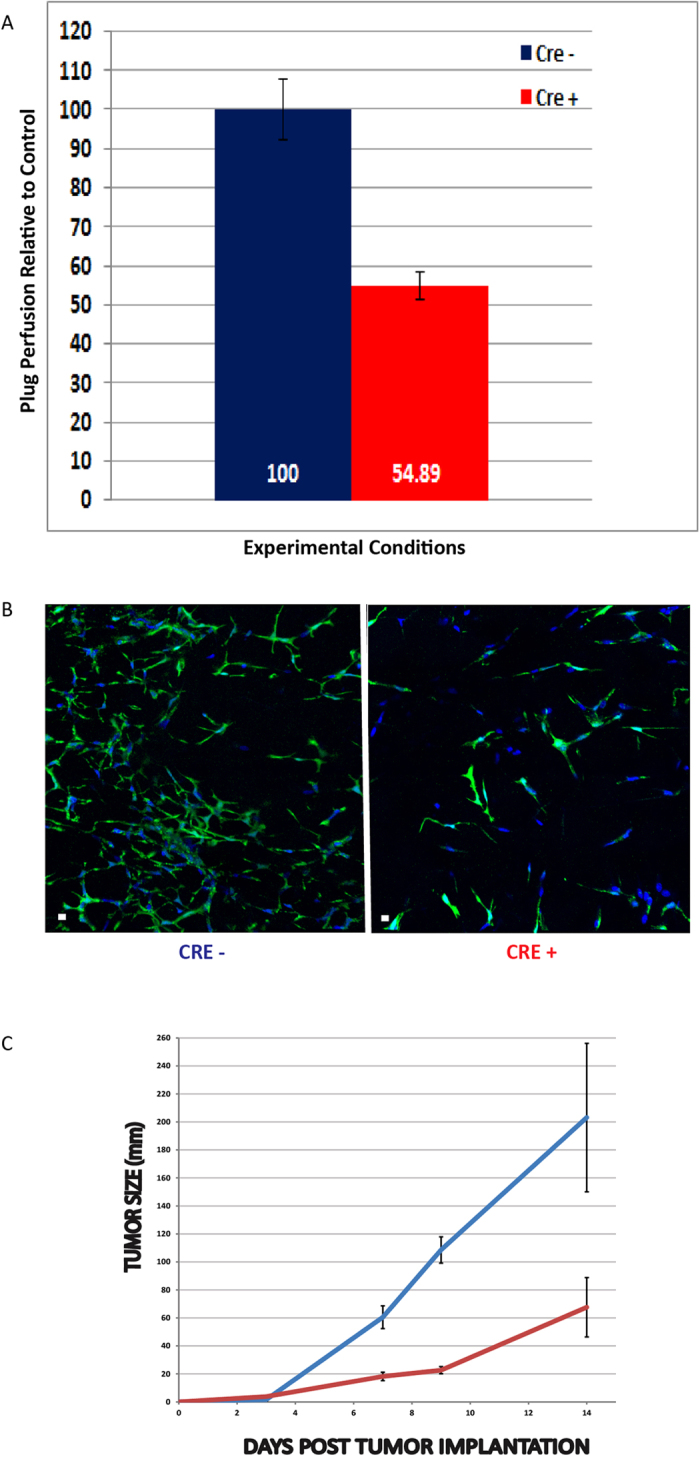
Hypoxia is an important driver of VM network formation. (**A**) ***LysM^Cre^*/HIF-1α^flox/flox^; Cx3cr1^GFP/+^** mice were injected subcutaneously with matrigel and 10 days later iv infused with 3 KD dextran. Dye was allowed to circulate for 5–7 minutes, mice euthanized, plugs harvested and the dye measured spectrophotometrically. Cre^+^ mice had 55% reduction in plug perfusion relative to control (Cre^−^ mice). The graph represents the combined results of 3 experiments. (**B**) Knockdown of myeloid specific HIF-1α significantly reduces CX3CR1^*GFP/*+^ cell infiltration into the plug and has a concomitant decrease in network formation relative to control. (**C**) ***LysM***^**cre**^**/HIF-1α** mice were implanted with B16/F10 melanoma cells and tumor size was measured every other day for 14 days. Cre^+^ mice had a 67% reduction in tumor size at 14 days relative to control. Two experiments were conducted with an N = 6–7 mice/group/experiment.

Since tubular network formation is an energy-dependent activity, we hypothesized that oxygen-regulating factors may be an important driver of this process and thus, chose to determine if network formation was driven by macrophage-specific expression of Hif-1α. To answer this question, LysM-Cre; HIF-1α^flox/flox^; CX3CR1^GFP/+^ mice were subcutaneously injected with matrigel and 10 days later intravenously infused with 3 KD rhodamine dextran. In this study, Cre^+^ mice had a 45% decrease in plug perfusion as well as a decrease in macrophage VM network formation relative to control mice (Fig. 6A,B).

With the result that hypoxia drives this process, we asked if network formation might also occur in other hypoxic environments such as tumors. As a first step, we tested the effect of myeloid-specific HIF-1α knockout on tumor growth. B16/F10 tumor cells were implanted in ysM-Cre; HIF-1α^flox/flox^; CX3CR1^GFP/+^ mice and tumors were allowed to grow. In this experiment, Cre^+^ mice had a 67% reduction in tumor growth compared with their HIF-1α Cre^−^ littermates 14 days after tumor cell inoculation (Fig. 6C).

### In tumors, macrophages form perfused VM channels

**Figure 7:**
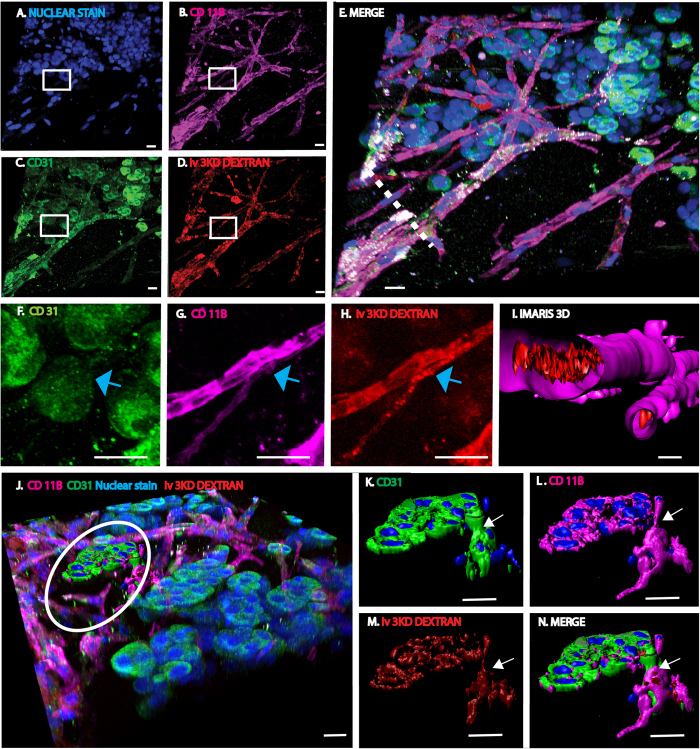
Macrophages form a perfused vascular network in tumors. (**A–D**) Images represent individual laser channels of a 38 *μm* Z-stack representing an A375 human melanoma subcutaneous tumor in an athymic mouse. Tumor cells were injected in the absence of matrigel and tumor perfusion was assessed by administering a 3 KD rhodamine dextran by tail vein injection prior to tumor removal. Tumor was harvested, fixed and immunostained with anti-mouse antibodies as follows: (**A**) Hoechst nuclear stain (blue), (**B**) CD11b (purple), (**C**) CD31 (green) and (**D**) iv 3 KD rhodamine dextran (red). (**E**) Merged image comprised of the four fluorescent channels presented in (**A–D**). Note that most of the perfused structures are strongly positive for CD11b. (**F–H**) Magnified view of individual laser channels in the area highlighted by the white box in (**A–D**) shows that the intravenously injected dye localizes to the CD11b^+^ tubular structure (blue arrow) which is not CD31^+^. (**I**) Imaris 3D cross sectional rendering of the CD11b^+^ vessels depicted by the dashed white line in image (**E**) shows the dye to be within the lumen of the CD11b^+^ channel. (**J–N**) CD11b^+^ tubes are connected to multinucleated, tuft-like structures which express CD31, CD11b and are perfused by systemically injected dye. (**J**) Low power view of a rosette-like structure within the tumor associated macrophage VM network. Circled area shows an Imaris rendered tumor tuft (**K–N**) that is connected to the predominately CD11b tubular network with a stalk-like structure (arrow). Tufts are multinucleated (blue) and express CD31 (**K**) (green), CD11b (**L**) (purple) and are perfused by systemically injected dye (**M**) (red). (**N**) Image of merged channels. Quantitative analysis showed that 25% of the cells detected were CD11b^+^ and associated with 61% of the dye. All scale bars are 20 um.

To determine if macrophages form channels within tumors, we studied 2 melanoma models. Initial immunohistochemistry experiments confirmed that neither cell line expressed the macrophage cell surface markers CD11b or F4/80 *in vitro* (data not shown). Mouse B16/F10 or human A375 melanoma cells were injected subcutaneously with or without matrigel into C57BL/6J or athymic mice, respectively. In both subcutaneous tumor models and with or without matrigel, cells expressing the macrophage markers CD11b, CD163 and F4/80 formed a network of interconnected cells in the tumor stroma (Fig. 7, SI. 9A–F) indicating that macrophages also formed VM networks in these tumor models. Similar to the network formed in the non-tumor subcutaneous matrigel model, the tumor-associated macrophage network also stained with endothelial markers CD31 and lymphatic marker LYVE1 (SI. 9G). To determine if the VM network connected to the pre-existing vasculature, a 3 KD rhodamine dextran was administered to tumor-bearing mice via tail vein injection immediately prior to euthanization and explantation of tumors. Similar to our findings in the non-tumor model, the tumor-associated macrophage network also filled with intravenously injected dye (Fig. 7, SI. 9D,E,H,I SI. movies 1 and 2). For example, in the representative image shown, 61% of the perfused dextran is associated with the CD11b^+^ surface area whereas only 33% of it is associated with CD31^+^ signal. These findings support the concept that macrophages can form vascular mimicry channels in tumors.

### Tumor tufts are perfused multinucleated structures

In both A375 and B16/F10 melanoma models there were multinucleated rosette-like structures, or tufts, arising from the macrophage VM network. Immunofluorescence analysis of tumor tissue showed that tufts strongly express CD31, as well as the macrophage markers CD11b, CD163 and F4/80 (Fig. 7J–N, SI. A–E). The tufts extended above the plane of the VM network via a stalk-like structure (Fig. 7K–N arrow). These structures, including the tuft itself, were functionally connected to the systemic circulation since they filled with tail vein-injected dyes (Fig. 7, SI. 9D,E,H,I). This observation indicated that tufts, in the images analyzed, are part of the VM network and most likely are constructed of macrophages and potentially ECs.

### A macrophage network is present in human tumors

To determine if this macrophage network is present in human tumors, we performed immunohistochemical analysis for the macrophage marker CD163 and the ‘endothelial’ marker CD31 on human tissue specimens from 5 skin melanomas, 2 spinal schwannomas, 1 cerebellar hemangioblastoma, 3 meningiomas (1 malignant WHO grade 3, 2 benign WHO grade 1), and 2 glioblastomas. In each of these tumor specimens, there was extensive CD163 staining. For in depth analysis, both benign and malignant meningiomas were studied. In multiple areas within the malignant meningioma specimen, a network of interconnected CD163^+^ cells (SI. 10B) superimposed with the highly patterned PAS^+^ network (SI. 10D) The PAS^+^ stain has been used by other investigators to delineate vascular mimicry channels^26^. However, in the areas of extensive macrophage network, CD31^+^ vessels were sparse (SI. 10C). In a benign meningioma specimen, antigen retrieval followed by immunohistochemistry for CD163 and Imaris rendering, revealed CD163^+^ multinucleated tubular structures surrounding clusters of meningioma cells (SI. 10E–G). The interconnected CD163^+^ tubules may represent vascular mimicry channels in a **benign** tumor.

## Discussion

In this study, we identify a novel role for macrophages. These cells are the key effectors of the innate immune response. However, they are also recognized to play a supportive role in wound healing and angiogenesis. Here we identify an additional structural role for them in the formation of a primitive tubular network in both subcutaneous matrigel and tumors. In both models, the conduits are connected to the systemic vasculature since intravenously injected dye fills the tubular network. Finally, myeloid-specific HIF-1α is an important mediator of the formation of the VM network in both matrigel and tumors since its ablation leads to a decrease in network formation, plug perfusion and tumor growth.

While the macrophage network shares features with those derived from arterial, venous and lymphatic endothelial cells, it differs in several ways. For example, the primitive conduits are perfused like arteries and veins, but the macrophage conduits lack clear tight junctions. Lymphatic endothelial vessels also share features with this macrophage network since the layered cell processes at cell junctions are similar and both lymphatics and the macrophage tubular networks do not appear to transport red blood cells. However, the collagen in the macrophage channels is found within the lumen while the collagen for all types of endothelial vasculatures is abluminal^27^. Therefore, this macrophage network shares features with all types of endothelium, but is distinct.

Anghelina *et al*.^13^ described how macrophages migrate into matrigel and form “branched cell columns”. They postulated that macrophages may influence the patterning of the eventual incoming blood vessels or potentially transdifferentiate into endothelial cells. In addition, other researchers have reported the transdifferentiation of monocytes/macrophages into either EC and EC-like cells *in vitro* and *in vivo*^13^,^15^,^16^ or mural cells^17^. Yet, our work does not address whether the macrophages forming the vascular mimicry conduits eventually transdifferentiate into EC or mural cells.

How does this data fit into the known paradigm of what constitutes a “vasculature?” One hypothesis is that macrophages respond first to hypoxic conditions within the subcutaneous plug by migrating and forming an interconnected network of cords that matured into tubes as a function of time. These conduits may provide nutrients to cells populating the plug during the vascular construction process. Potentially, ECs invade the tubes displacing the collagen and relocating it to the external surface of the developing blood vessel. The macrophages that initially formed the lumen would now “hug” the abluminal surface of the vessel in the appropriate location where a perivascular macrophage or mural cell is commonly found.

Although our initial work was done in subcutaneous matrigel, these VM channels were also observed in tumor models without matrigel. In a subcutaneous tumor model, we found macrophage-lined VM channels in early stages of tumor growth. Yet beyond the 7–10 day stage, particularly using B16/F10 tumor cells, imaging is impeded by tissue thickness and melanin deposition. However, in human tumor specimens we identified CD163^+^ macrophage networks that coincide with PAS^+^ stain. These PAS^+^ regions form “looping” networks which identify vascular mimicry channels in human tumors^6^. Furthermore, Caillou *et al*., also identified an interconnected macrophage network in multiple cases of anaplastic thyroid^28^. Lastly, another recent discovery is the presence of circulating multinucleated giant macrophages complexed with tumor cells in the blood of cancer patients^29^. These giant cells express both macrophage and endothelial markers. One possibility is that macrophages forming the VM channels may be shed into the bloodstream and contribute to the circulating giant macrophage-like population.

A characteristic feature of the subcutaneous tumor macrophage network is the multinucleated structures (tufts) which are also perfused by dye after intravenous administration. Although pinocytosis of dye by macrophages is possible within the 5–15 min between injection and tissue fixation, a substantial role for phagocytosis or pinocytosis of dye is unlikely given the clear pattern of dye within the conduits and tufts and the absence of extracellular dye. Furthermore, intravenous injection of dye followed by its appearance within a tubular network supports the idea that it is connected to the systemic circulation. However whether the vessels fill through capillary action or cardiac action is not clear. It is also not clear whether the conduits form a plexus that reconnects back with systemic circulation versus an end-artery tree-like configuration as with the vasa vasorum^30^.

Non-endothelial vasculatures have been previously described. Moldovan *et al*., discovered that macrophages drill channels in the ischemic heart in a hypoxic process that resembles the one described here^3^. Another form of non-endothelial vasculature is found in tumors. Cancer stem cells are believed to form vascular mimicry channels that express various markers including CD34, CD44, and CD133 as well as PAS^+^ staining^31^. Yet, in our work, the VM channels in both tumor and non-tumor models are formed by macrophages. Furthermore, we and other groups have also shown that macrophages can express these “cancer stem cell markers”^32^,^33^,^34^.

In subcutaneous matrigel, macrophages form a three dimensional tubular network through a metabolically demanding process that’s mediated in part by myeloid-specific expression of HIF-1α. Supporting our results, Ahn *et al*. found that HIF-1α activation in monocytes was the primary effector of vessel formation within the matrigel plug^35^. Matrigel plugs are approximately a square centimeter in size which is well outside the 100 μm range for diffusible oxygen^36^. This suggests that this process most likely requires glycolysis. The primitive vasculature in both matrigel and tumors may deliver some of the nutrients needed to support this process.

Initial experiments with different molecular weight dyes showed that lower MW dyes (3–10 KD) readily accessed the tubular network, but larger dyes did not. In humans, gadolinium DTPA (0.938 KD) is a low molecular weight MRI contrast agent that is injected intravenously to identify tumors by their bright enhancement on MRI scans. The tumor enhancement is believed to represent spillage of dye from leaky blood vessels in malignant tumors. Our data suggests that low molecular weight molecules including gadolinium and glucose, may flow from the blood vessels into the macrophage-lined conduits further dispersing the dye within the tumor.

Interestingly, our limited study of benign tumors (meningiomas and schwannomas) in humans identified a macrophage network similar to that observed in malignant tumors. To our knowledge, vascular mimicry channels have not been identified in benign tumors. Yet, these benign tumors often brightly enhance with gadolinium by MRI^37^ despite a generally reduced blood supply and slower growth rate than that of malignant tumors. In addition to tumor-associated endothelial vasculature, we postulate that the extent of gadolinium enhancement of benign and malignant tumors may reflect the contribution of these macrophage mimicry channels to tumor perfusion.

From an evolutionary perspective, this non-endothelial “vasculature” shares features with the circulatory system of many invertebrates. In general, invertebrate vasculature is not lined by endothelial cells, oxygen is carried by plasma proteins rather than by RBCs, and the vessels have basement membrane proteins within the lumen rather than on the abluminal surface as found in vertebrates. In the invertebrate amphioxus, phagocytic cells - the ancestral relatives of macrophages - are believed to form the circulatory tubes by hollowing out the ECM^4^. As proposed by Kucera and Lammert, this ancestral process may be still relevant under certain conditions in vertebrates, particularly in the vascular mimicry channels of certain tumors^38^. While this process has been mainly attributed to cancer stem cells, here we show that the vertebrate macrophage maintains the ability to form primitive vascular conduits.

Recently, macrophages have been implicated in limb regeneration in the salamander^39^. Ablation of macrophages with clodronate prevents limb regeneration. However, the process is reversible. With restoration of macrophages, the salamander regains the ability to regenerate limbs. Our finding of the fundamental role of macrophages in hypoxic angiogenesis offers a potential explanation: with macrophage depletion, the initial macrophage “vasculature” may not form within the hypoxic tissues of the amputated limb, preventing angiogenesis and limb regeneration.

Endothelial cells have been considered responsible for essentially all types of vascular channels including those which are formed under hypoxic conditions^4^,^5^,^40^. Here we present evidence that the macrophage, an immune cell, plays a role in creating vascular mimicry channels under hypoxic conditions as seen in cancer. This alternate form of vasculature provides additional therapeutic targets for rational drug and treatment design.

## Materials and Methods

### Animal Lines

Wild type C57BL/6J and Athymic nude mice were obtained from The Scripps Research Institute’s Rodent Breeding Colony. Also, transgenic mice in which either one (CX3CR1^GFP/+^) or both (CX3CR1^GFP/GFP^) copies of the CX3CR1 gene were interrupted by the enhanced green fluorescent protein gene (eGFP). Mice expressing Cre recombinase under the LysM promoter (LysM-Cre mice; were mated with HIF-1α^flox/flox^ or R26^iDTR/+^ 41. Genotyping was performed by Transnetyx, Inc. (Memphis, TN). Mice were housed under standard conditions in an animal facility at The Scripps Research Institute and care was provided in accordance with institutional guidelines. All experiments involving experimental animals were performed in accordance with the NIH Guide for the Care and Use of Laboratory Animals and all procedures were approved by The Scripps Research Institute Animal Care and Use Committee.

### Cell Lines

B16/F10 (mouse melanoma) and A375 (human malignant melanoma) cells were purchased from the American Type Culture Collection (Manassas, VA) and cultured in DMEM media (4 mM L-glutamine, 4500 mg/L glucose, 1 mM sodium pyruvate and 1500 mg/L sodium bicarbonate) supplemented with 10% Fetal Bovine Serum and 1X Penicillin-Streptomycin (Gibco/Life Technologies, cat no. 10437028 and 10378016 respectively). Cells were maintained *in-vitro* at 37 °C in a humidified 5% CO_2_ incubator.

### *In vivo* subcutaneous angiogenesis Assay

Growth factor reduced matrigel (Becton Dickinson, cat no. 356230 or 354230) was stored at −20 °C, thawed overnight at 4 °C and supplemented with either mouse recombinant IFN-γ (R&D Systems, cat no. 485-MI-100) or Human recombinant VEGF165 (Peprotech, cat no. 100–20) at 2 μg or 400 ng per plug respectively or saline. Pre-chilled pipet tips, stripettes and 5 ml falcon polypropylene tubes were used during matrigel preparation. Experimental mice were anesthetized with an intraperitoneal injection of 40 μl Ketamine (50 mg/ml)/Xylazine (5 mg/ml), their right side was shaved, wiped with 70% isopropyl alcohol and 450 μl of chilled matrigel solution was subcutaneously injected into the right posterior flank using a pre-chilled 1 ml 27G-needle syringe (Becton Dickinson, cat no. 305620). Matrigel remains liquid at 4 °C but forms a solid gel at 37 °C, therefore trapping the cytokine(s) to allow for slow release. Between 30 minutes and 14 days following matrigel injection, mice were euthanized, subcutaneous plugs harvested and fascia surrounding the plug was carefully removed in order to strictly evaluate the environment within the plug while excluding the external connective tissue. Subcutaneous matrigel plugs were then evaluated for perfusion, sectioned and stained for EM, immunohistochemistry or enzymatically digested to isolate the cells for flow cytometry. In all experiments at least 3–8 mice were used per experimental condition or time point. All experiments were repeated 2–3 times. The time course experiment was performed in its entirety one time but results at different time points were confirmed within different experiments.

### Plug Perfusion Assay

Mice were intravenously injected (tail vein) with 200 μl of a 10 mg/ml, anionic, Lysine fixable, 3,000 MW Fluorescein or Rhodamine conjugated Dextran (Life Technologies, cat no. D3306 and D3308 respectively, Carlsbad, CA). Dextran was allowed to circulate for 7 minutes at ambient temperature before mice were euthanized and plugs harvested. Dissected plugs were placed on ice, weighed and homogenized in 750 μl of chilled RIPA buffer (Sigma, cat no. R0278) for 1 min at room temperature in the dark. Solutions were allowed to settle in the dark for 1 hour at 4 °C and 100 μl of plug solution, in triplicate, was transferred to a 96 well, black sided with clear bottom assay plate (Corning Costar, cat no. 3603) and read in a BioTek Synergy plate reader. Dissections and spectrophotometric analysis were performed in a blinded fashion.

### Immunofluorescence

Harvested plugs were washed with cation-free PBS (Gibco, cat no. 14190144) and fixed overnight at 4 °C in 4% PFA in PBS. Next day, plugs were washed with 1 × PBS, casted in 5% Agarose/H20 (Invitrogen, Cat no. 16500–100) and 200–300 μm tissue sections were prepared with a Leica Vibratome model VT-1000P. In order to block any nonspecific binding, vibratome sections were blocked overnight at 4 °C with 5% goat, 5% donkey serum in PBS. Next day, sections were washed in PBS and incubated overnight at 4 °C with the desired primary antibodies (1:100). We used rat mAbs against mouse CD31 (BD Pharmingen, cat no. 550274), CD163 (Abcam, cat no. ab119996), CD11b (eBioscience, cat no. 14-0112), F4/80 (eBioscience, cat no. 14-4801), CD206 (AbD Serotec, cat no. MCA2235), CD34: (LifeSpan Biosciences, cat no. LS-C62600), Rat IgG2b, k Isotype control (eBioscience, cat no. 14-4031), polyclonal Goat Anti-Mouse Ab AQP4 (Santa Cruz, cat no. sc-9888) and polyclonal Rabbit Abs against Collagen Type IV (Millipore, cat no. AB756P), LYVE-1 (Abcam, cat no. ab14917), ADAM17 (Millipore, cat no. AB19027), MMP-9 (Millipore, cat no. AB19016), CD206 (Abcam, cat no. ab64693), CD31 (Abcam, Cat no. ab28364) and Phospho-p44/42 MAPK (Cell Signaling, cat no. 9101). Tissue sections were then washed 3X in PBS prior to 4 °C overnight incubation with the corresponding Alexa Fluor-conjugated secondary antibodies (Invitrogen, 1:400). Next day, sections were washed 3X with PBS and nuclei stained with Hoechst 33342 (Invitrogen, cat no.H3570) for 15–20 minutes at room temperature in the dark. Sections were washed 3X with PBS and mounted on poly-L-Lysine coated frosted slides (Electron Microscope Science, cat no. 2951-006) with a drop of slowfade reagent (Life Technologies, cat no. S36937). Appropriate no secondary controls were performed in all experiments.

### Cell isolation from matrigel plug, flow cytometry and RNA isolation

Subcutaneous matrigel plugs were digested for 60–90 minutes at 37 °C in an enzymatic cocktail composed of 25 μg/ml DNase-I, grade II (Roche, cat no. 10-104-159-001), 3 U/ml Dispase (Roche, cat no. 10-269-638-001), 3 U/ml Liberase TM Research grade (Roche, cat no. 05-401-119-001) and 25 μg/ml Hyaluronidase Type IV-S (Sigma, cat no. H3884). The isolated cells were filtered through a 70 μm Nylon cell strainer (BD Falcon, Bedford, MA) into a sterile 50 ml centrifuge tube and cells washed 3X with FACS Buffer (1X PBS without Ca/Mg, 2 mM EDTA and 0.5% BSA).

Isolated cells were analyzed by flow cytometry to determine their cellular characteristics. FITC-conjugated F4/80 (eBioscience, cat no.11-4801) and APC-conjugated CD31 (eBioscience, cat no. 17-0311) were used to distinguish endothelial cells from macrophages with additional labeling using eFluor450-conjugated CD34 (eBioscience, cat no. 48-0341), PE-conjugated CD-133 (eBioscience, cat no. 12-1331) or PERC-P conjugated CD45 (eBioscience, cat no. 45-0451). Antibodies were incubated with isolated cells in FACS buffer for 30 minutes at 4 °C in darkness. Cells were washed 3X with FACS buffer and fixed in 1% PFA in PBS for 10 minutes at room temperature in the absence of light. Fixed cells were washed in PBS and resuspended in 400 μl of FACS buffer for flow cytometry. Experiment performed three times.

### Transcriptomic Arrays

Total RNA was prepared from FACS sorted F4/80^+^ cells isolated from subcutaneous matrigel plugs using the RNeasy Plus Mini kit (Qiagen). RNA was reverse transcribed using RT^2^ First Strand cDNA Kit (Qiagen) and the expression of 84 genes known to modulate the process of angiogenesis were analyzed according to the manufacturer’s instructions using the Mouse Angiogenesis RT^2^ Profiler PCR Array (Qiagen, Cat no. PAMM-024 and PAMM-024C). Quantitative PCR assays were performed on a real-time PCR System (ABI 7900HT Fast, Life Technologies). Experiment performed twice using two different PCR Arrays.

### Subcutaneous Tumor Model

- Subconfluent B16/F10 murine melanoma cells were harvested with 4 mls of TrypLE Express (Gibco, cat no. 12605-010) per T75 for 3 minutes at 37 °C in a humidified 5% CO_2_ incubator. Trypsin activity was stopped with 8 mls of complete media per T75 and cells centrifuged at 1,100 RPM for 5 minutes at room temperature. Cells were washed with serum free media and resuspended in unsupplemented Dulbecco’s Modified Eagle medium (DMEM). LysM-cre(+); HIF-1α^flox/flox; Cx3cr1GFP/+^ or HIF-1αflox/flox; Cx3cr1^GFP/+^ mice were injected subcutaneously with 2.5 × 10^5^ B16/F10 cells in 50 μl of serum-free DMEM or in 450 μl of growth factor reduced matrigel in the right posterior flank^42^. Tumors were allowed to grow for 14 days while size was monitored every other day with calipers. Tumor sizes were estimated using the formula A = L × W, where A = area, L = length, and W = width. Three, seven, ten and fourteen days post tumor cell injection, mice were intravenously injected (tail vein) with 200 μl of a 10 mg/ml dextran–tetramethylrhodamine-saline solution (3,000 MW, Anionic, Lysine Fixable, Life Technologies, Cat no. D3308) and dextran was allowed to circulate for 7 minutes before mice were euthanized and subcutaneous tumors harvested. B16/F10 tumors were fixed overnight at 4 °C in 4% PFA in PBS. Next day, tumors were washed in PBS and casted in 5% Agarose (Invitrogen, cat. no.16500-100). Tumor sections (250 to 300 μm) were cut with a Leica vibratome and sections were processed as those previously described for subcutaneous matrigel plugs.
- Similarly, A375 human melanoma cells were harvested, washed, and resuspended in unsupplemented DMEM as previously described for B16/F10 cells. Athymic nude mice were injected subcutaneously in their right posterior flanks with 2.0 × 10^6^ A375 tumor cells in either 50 μl of serum-free DMEM or in 450 μl of growth factor reduced matrigel. Three and five days after tumor cell injections, when small palpable tumors were detected, mice were intravenously injected (tail vein) with 200 μl of a 10 mg/ml dextran–tetramethylrhodamine-saline solution (Life Technologies, Cat no. D3308) and dextran was allowed to circulate for 7 minutes before mice were euthanized and subcutaneous tumors harvested. A375 subcutaneous tumors were processed as described above.

### Subcutaneous matrigel plug perfusion and c-Fms inhibitor or clodronate treatment

C56BL/6J mice were anesthetized with Ketamine/Xylazine, shaved, right flanks swabbed with alcohol and subcutaneously injected with 450 μl of chilled growth factor reduced matrigel solution (supplemented with VEGF or IFN-γ) in the right posterior flank. A c-Fms tyrosine kinase inhibitor, Tocris Ki20227 (R&D Systems, cat no. 4481) was dissolved in DMSO at 50 mg/ml and injected i.p. at a dose of 20/mg/kg/day (25 g mice) for the duration of the experiment. For Clodrosome (liposomal clodronate) treatment, 200 μl of a 5 mg/ml Clodrosome solution (Encapsula Nano Sciences) was injected i.p. into adult mice every 48 hours for the duration of the experiment. Encapsome (control Liposome Suspension) was used as control and injected daily (200 μl per mouse of a 5 mg/ml solution). Both Ki20227 and Clodrosome treatments commenced 24 or 48 hours prior to subcutaneous matrigel injections. Experiments were performed in a blinded fashion 2–3 times for all conditions.

### EM

Matrigel plugs were removed from the host animal and carefully cleaned of superficial connective tissue before being immersed and fixed overnight in ice cold 2.5% glutaraldehyde in 0.1 M Na cacodylate buffer (pH7.3). Once firm, the Matrigel plugs were further chopped into smaller pieces and continued fixing a second night in the buffered glutaraldehyde. Treatment of the samples continued with a buffer wash, additional fixation in buffered 1% osmium tetroxide and buffer washed again. After a brief wash in distilled water, the Matrigel pieces were dehydrated in graded ethanol series, transitioned in propylene oxide and embedded in Embed 812/Araldite (Electron Microscopy Sciences, Hatfield PA). Thick sections (2 μm) were cut, mounted on glass slides and stained in toluidine blue for general assessment in the light microscope. Subsequently, 70 nm thin sections were prepared, mounted on copper slot grids coated with parlodion and stained with uranyl acetate and lead citrate for examination on a Philips CM100 electron microscope (FEI, Hillsbrough OR). Images were documented using a Megaview III ccd camera (Olympus Soft Imaging Solutions GmbH, Münster, Germany). Multiple experiments were performed at multiple time points as described in manuscript.

### Confocal Microscopy, Analysis and Quantification

All images were gathered with a confocal laser-scanning microscope (LSM 700 or 710, Carl Zeiss) utilizing a Plan-Apochromat 20X/0.8, Plan-Apochromat 63X/1.4 Oil DIC, C- Apochromat 40X/1.2W Korr UV-VIS objective lens (Carl Zeiss) and processed with the ZEN 2010 software (Carl Zeiss). Scanning was performed in sequential laser emission mode to avoid scanning at other wavelengths. Three-dimensional reconstructions were generated using ZEN 2010 and Imaris software (BitPlane, South Windsor, CT). Z-stacks were acquired using a Zeiss 710 laser scanning confocal microscope using a 20x objective (1 μm step size), or a 63x objective (0.3 μm step size) and assembled in the Zen software. Raw images were imported into both Imaris (Bitplane) and Image Pro Premier (IPP) (Media Cybernetics) where they were further processed for total volume, area and cell counts. Using Imaris, each of the channels composing the Z-stack were iso-surfaced using the Surpass module in order to create a 3 dimensional representation of the image. Furthermore, depending on the analysis performed, iso-surfaces were masked to define in 3 dimensions the signal in one channel and extract the signal from a second channel in order to determine how much signal resided within the mask created by the first signal. A similar and corroborating approach was used using IPP in 2D of maximum intensity projected (MIP) stacks. Here we outlined and masked total area of red dye and defined the amount of CX3CR1-GFP, CD11b or CD31 signals within this masked outline. Using the Hoescht (nuclear) labeling we were able to extract the numbers of cells with the masked 3D region in IMARIS and 2D regions in IPP. Results are shown as percent area or counts in figure legends.

## Additional Information

**How to cite this article**: Barnett, F. H. *et al*. Macrophages form functional vascular mimicry channels *in vivo*. *Sci. Rep.* **6**, 36659; doi: 10.1038/srep36659 (2016).

**Publisher’s note:** Springer Nature remains neutral with regard to jurisdictional claims in published maps and institutional affiliations.

## Supplementary Material

Supplementary Information

Supplementary audio

Supplementary audio

Supplementary audio
