# Supplementary material for: Macrophages form functional vascular mimicry channels in vivo
Source: Sci Rep. 2016 Nov 11;6:36659. doi: 10.1038/srep36659 (PMC5105153; doi:10.1038/srep36659)
Supplement: Supplementary Information [file srep36659-s1.pdf]

Macrophages form functional vascular mimicry channels in vivo

Authors: *Faith H. Barnett, *Mauricio Rosenfeld, Malcolm Wood, William Kiosses, Yoshihiko Usui, Valentina Marchetti, Edith Aguilar, and Martin Friedlander[1]

SI Figures and Legend

SI . 1

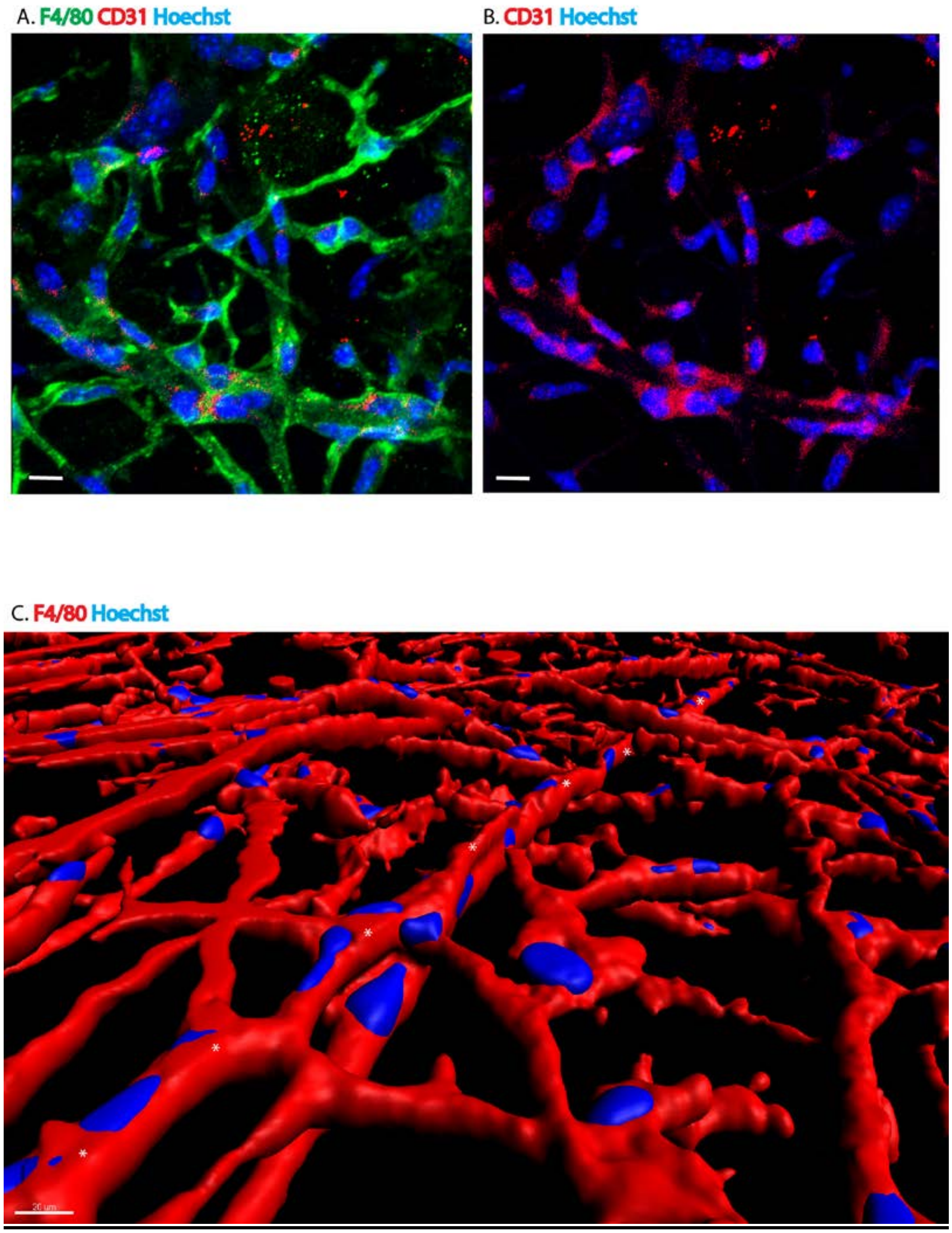

<u>SI.1 Macrophages form a multinucleated network.</u> All images are Z-stacks. (**A-B**) Immunohistochemistry for F4/80 (green) and CD31 (red) performed on sc matrigel plugs isolated from wild type (C57BL/6J) mice 6 days after matrigel injection. F4/80^{+} cells form a network of interconnected cells and some express CD31. Blue indicates Hoechst nuclear stain. These are mirror mages of Fig.1 A with the original color channels. (**C**) Imaris 3D rendering of a portion of Fig.1 G. An F4/80^{+} tubule (asterisks) extends over 200um. F4/80 (red) Hoechst nuclear stain (blue). All scale bars 20um.

SI. 2

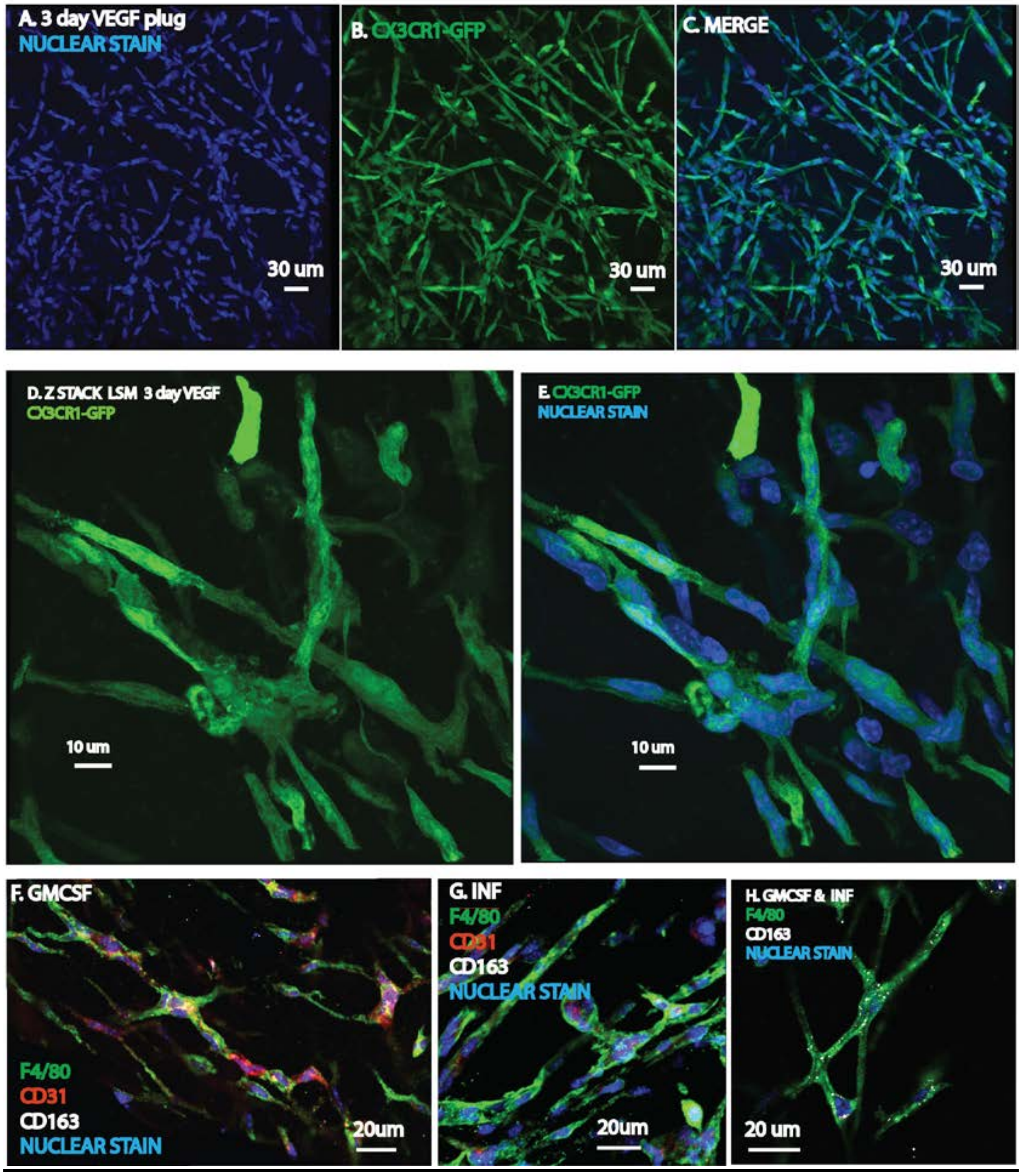

SI.2 Macrophage network forms in Matrigel with both VEGF and proinflammatory cytokine supplementation. All images are Z-stacks. (**A-E**) 2 channel confocal photomicrograph of a Z-stack taken of 3 day plug with VEGF supplementation implanted in CX3CR1$^{GFP/+}$ mice. (**A**) nuclear stain, (**B**) CX3CR1$^{GFP/+}$ and (**C**) merged channels. A dense CX3CR1$^{GFP/+}$ network is formed. (**D-E**) Close up view of CX3CR1$^{GFP/+}$ cells forming cord-like structures. (**F-H**) Ten day sc plugs supplemented with either GM-CSF (**F**), Interferon-γ (**G**), or GM-CSF and Interferon γ (**H**). A network is formed by F4/80^{+} (green) macrophages under pro-inflammatory cytokine stimulation. F4/80 (green), CD31 (red), CD163 (white) and nuclear stain (blue). Scale bars as indicated.

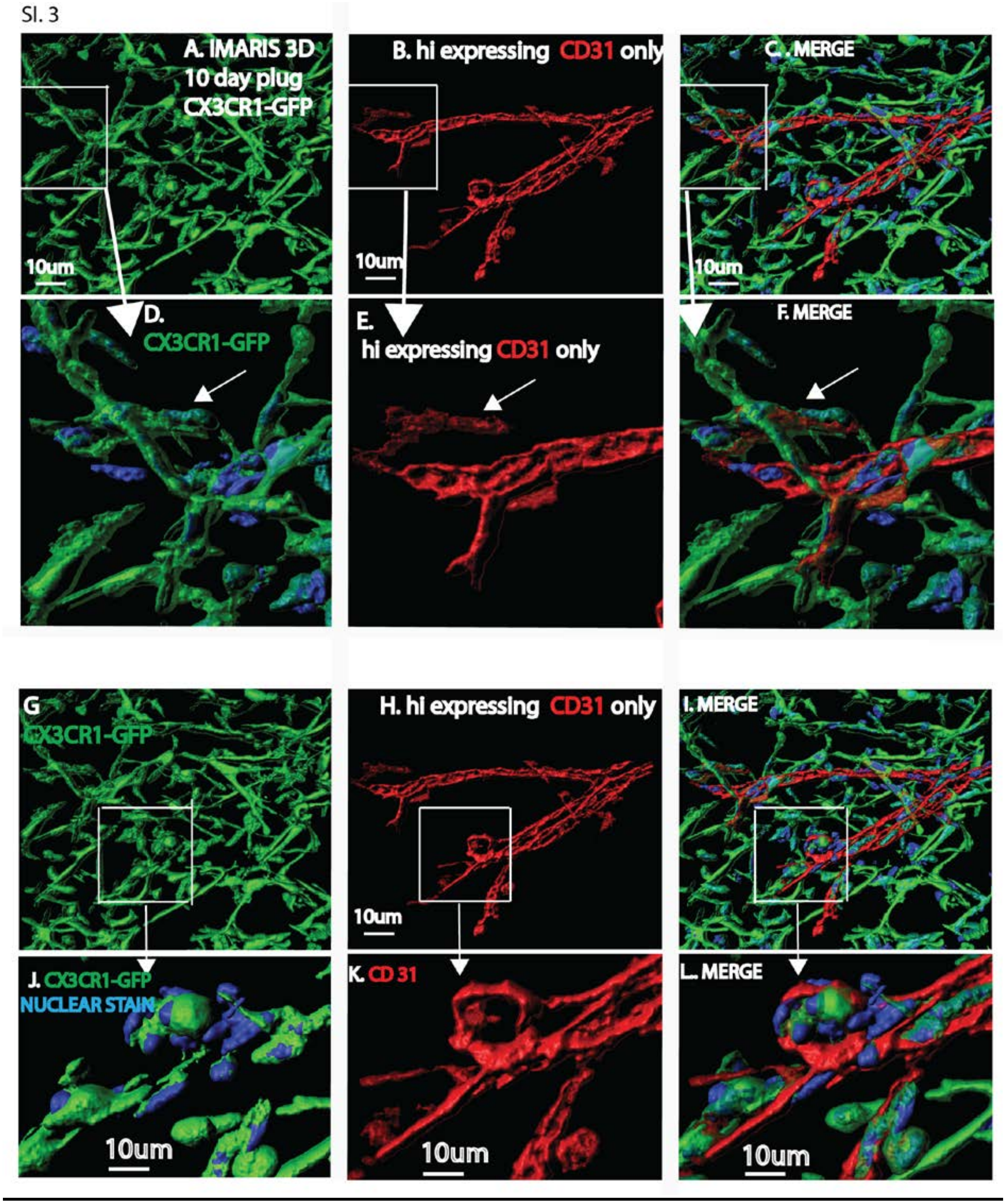

SI.3 Monocytes/Macrophages interact with the leading front of an invading blood vessel. (**A-C**) Further analysis of Figure 2 (**J-L**). (**D-F**) Magnified image of boxed area in (**A-C**). The CX3CR1$^{GFP/GFP}$ cells form a cuff around CD31 expressing tissue (white arrow). (**G-I**) depicts another area of interest at the leading front of the CD31 exprssing blood vessel. (**J-L**) A cluster of CX3CR1$^{GFP/GFP}$ cells are located at the leading front of the blood vessel; just above the plane of CD31 expressing filopodia. Scale bars as indicated.

SI. 4

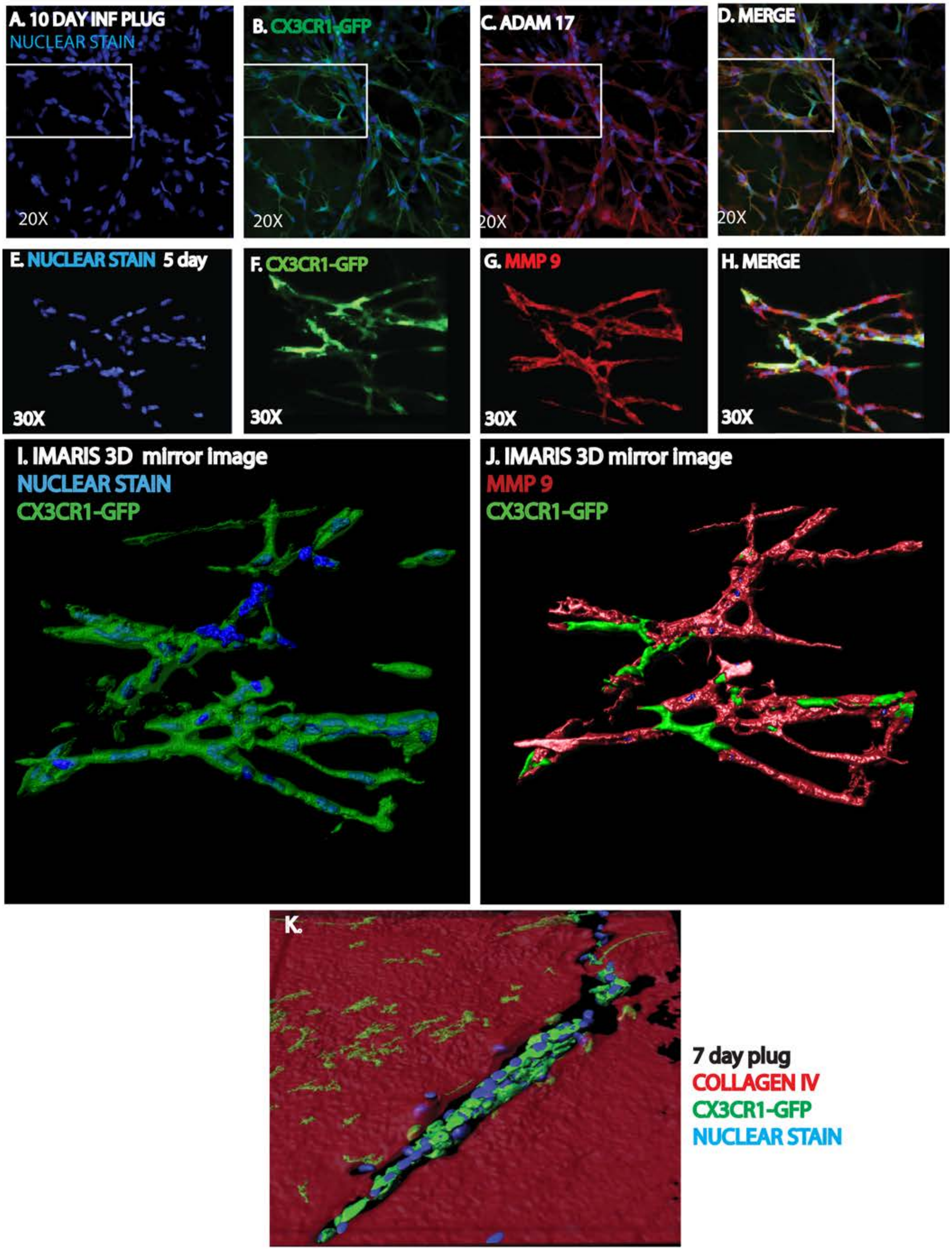
A. 10 DAY INF PLUG
NUCLEAR STAIN
20X
B. CX3CR1-GFP
20X
C. ADAM 17
20X
D. MERGE
20X
E. NUCLEAR STAIN 5 day
30X
F. CX3CR1-GFP
30X
G. MMP 9
30X
H. MERGE
30X
I. IMARIS 3D mirror image
NUCLEAR STAIN
CX3CR1-GFP
J. IMARIS 3D mirror image
MMP 9
CX3CR1-GFP
K.
7 day plug
COLLAGEN IV
CX3CR1-GFP
NUCLEAR STAIN

SI.4 CX3CR1$^{GFP/GFP}$ cells express ADAM17 and MMP9 between 2 hours and 10 days. (**A-D**) Immunohistochemistry analysis showing ADAM17 positivity on a 10 day subcutaneous matrigel plug isolated from CX3CR1$^{GFP/GFP}$ mouse. ADAM17 (red) and nuclear stain (blue). Invading CX3CR1$^{GFP/GFP}$ cells express ADAM17 in the subcutaneous matrigel plug. White box demarcates an area that is magnified in Figure 1**B**. (**E-H**) A five day matrigel plug was immunostained for MMP9. Individual channels are shown as indicated. (**I, J**) IMARIS 3D renderings of **E-H** but shown as mirror images. MMP9 is expressed by this CX3CR1$^{GFP/GFP}$ multinucleated tubular structure. (**I**) The green channel is rendered slightly translucent to allow visualization of the nuclei (blue). (**J**) Only the green and red channels are shown. (**K**) IMARIS 3D rendering of a 7 day subcutaneous matrigel plug from a CX3CR1$^{GFP/GFP}$ mouse. A multinucleated cord/tubular structure composed of CX3CR1$^{GFP/GFP}$ cells is found within a channel devoid of collagen IV. All images are Z-stacks.

SI. 5

RT-PCR angiogenesis array of RNA isolated from F4/80+ cells

Gene	Cycle	Gene	Cycle	Gene	Cycle	Gene	Cycle
Akt1	24.51	Il6	25.5	Pecam1	28.47	Angpt1	32.31
PPC	15.78	Mapk14	25.55	S1pr1	28.49	Pgf	32.4
PPC	16.25	Nrp2	25.81	Vegfb	28.98	Vegfc	32.6
Actb	18.24	Serpinf1	26.2	Epas1	29.07	Lect1	32.97
B2m	18.26	Itgav	26.34	Jag1	29.45	Pdgfa	34.05
Cxcl2	20.39	Thbs2	26.5	Ptgs1	29.54	Fgf1	36.87
RTC	20.59	Igf1	26.57	Fgf2	29.56	Ang	Undetermined
Ccl2	20.75	Anpep	26.67	Smad5	29.57	Bai1	Undetermined
RTC	20.94	Eng	26.69	Kdr	29.75	Col4a3	Undetermined
RTC	20.96	Tnfsf12	26.77	Tek	30.38	Erbb2	Undetermined
Tnf	21.57	Serpine1	26.83	Cdh5	30.49	F2	Undetermined
Gapdh	21.63	Edn1	26.85	Itgb3	30.53	Fgf6	Undetermined
Hsp90ab1	21.98	Ctgf	26.96	Timp1	30.54	Fgfr3	Undetermined
Il1b	22.66	Mmp19	27.02	Efnb2	30.78	Ifng	Undetermined
Plau	23.01	Mmp9	27.35	Csf3	30.88	Lep	Undetermined
Tgfb1	23.44	Figf	27.38	Tie1	30.94	Nos3	Undetermined
Timp2	23.63	Vegfa	27.45	Angpt2	31.11	Plg	Undetermined
Nrp1	24.35	Mmp2	27.51	Ephb4	31.32	Sphk1	Undetermined
Cxcl1	24.4	Ptk2	27.52	Mdk	31.35	Tbx1	Undetermined
Fn1	24.71	F3	27.89	Tgfb3	31.39	Tgfa	Undetermined
Gusb	25.25	Hgf	27.98	Col18a1	31.55	Tgfb2	Undetermined
Thbs1	25.27	Flt1	28.09	Cxcl5	31.73	Tymp	Undetermined
Hif1a	25.38	Mmp14	28.21	Egf	31.73	MGDC	Undetermined
Ccl11	25.43	Tgfbr1	28.46	Efna1	32.2	Ang	Undetermined
						Housekeeping Genes	

SI.5 PCR Angiogenesis array data. Three day subcutaneous matrigel plugs were harvested from C57BL/6J (wildtype) mice, dissociated and cells isolated. Cells were immunostained for F4/80 and subsequently sorted by FACS. RNA was isolated and RT-qPCR was performed using an Angiogenesis Array.

SI. 6

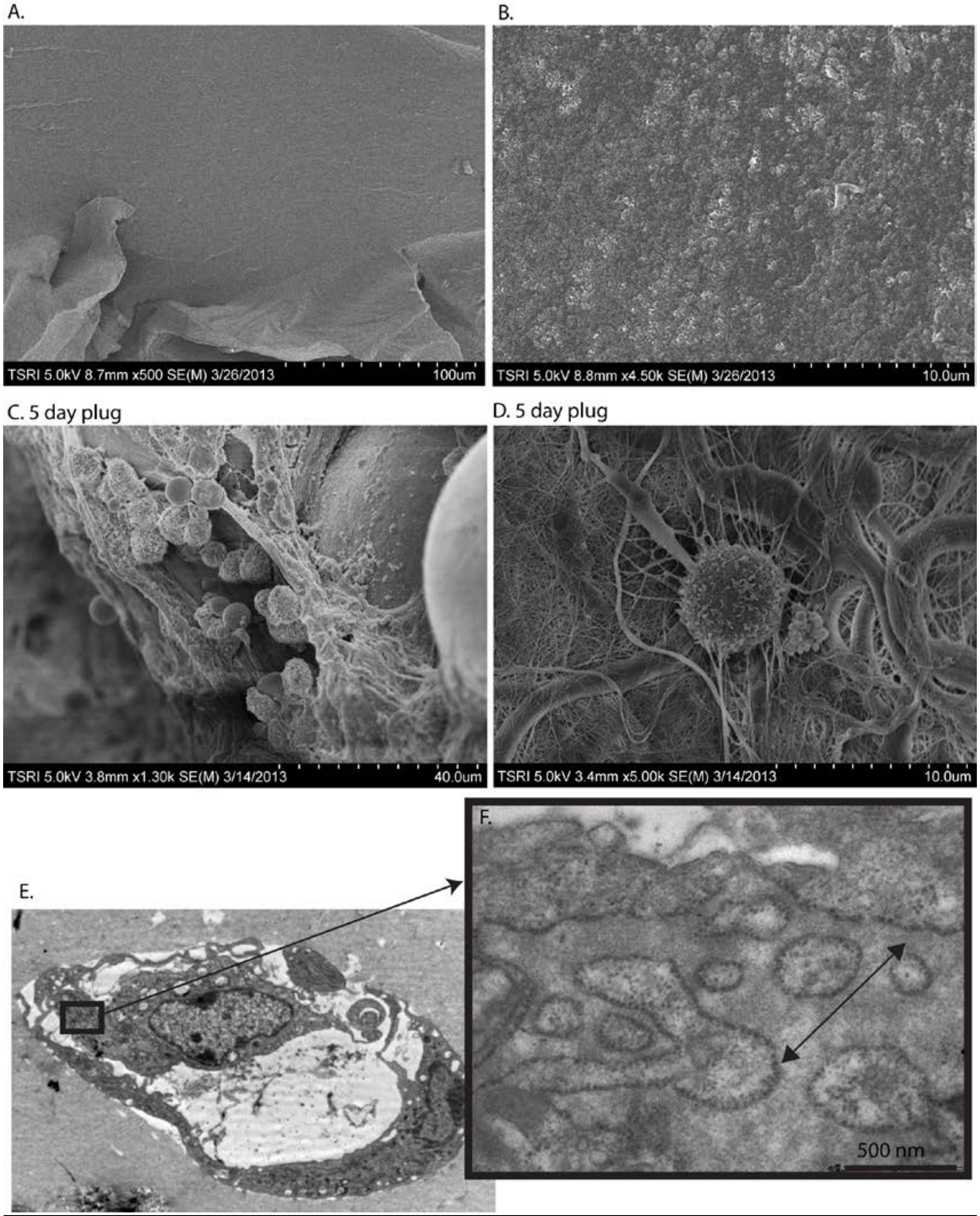

<u>SI.6 Electron microscopy of matrigel plug.</u> (**A,B**) Scanning EM image of matrigel solidified at 37°C on a plastic surface. (**B**) This figure is a 10X image of the plug shown in (**A**) and scale bar is bottom right. Note the absence of channels and cells. (**C,D**) Scanning EM of subcutaneous matrigel plug harvested from a mouse 5 days after matrigel injection. Note the presence of cells. (**E**) Transmission EM of cells inside an 11 day sc matrigel plug. Cells are justaposed within a cleared zone of matrigel. (**F**) Boxed area in (**E**) shows a markedly dilated rough endoplasmic reticulum (RER). Doubled-headed arrow shows lumen of RER. Scale bars as shown.

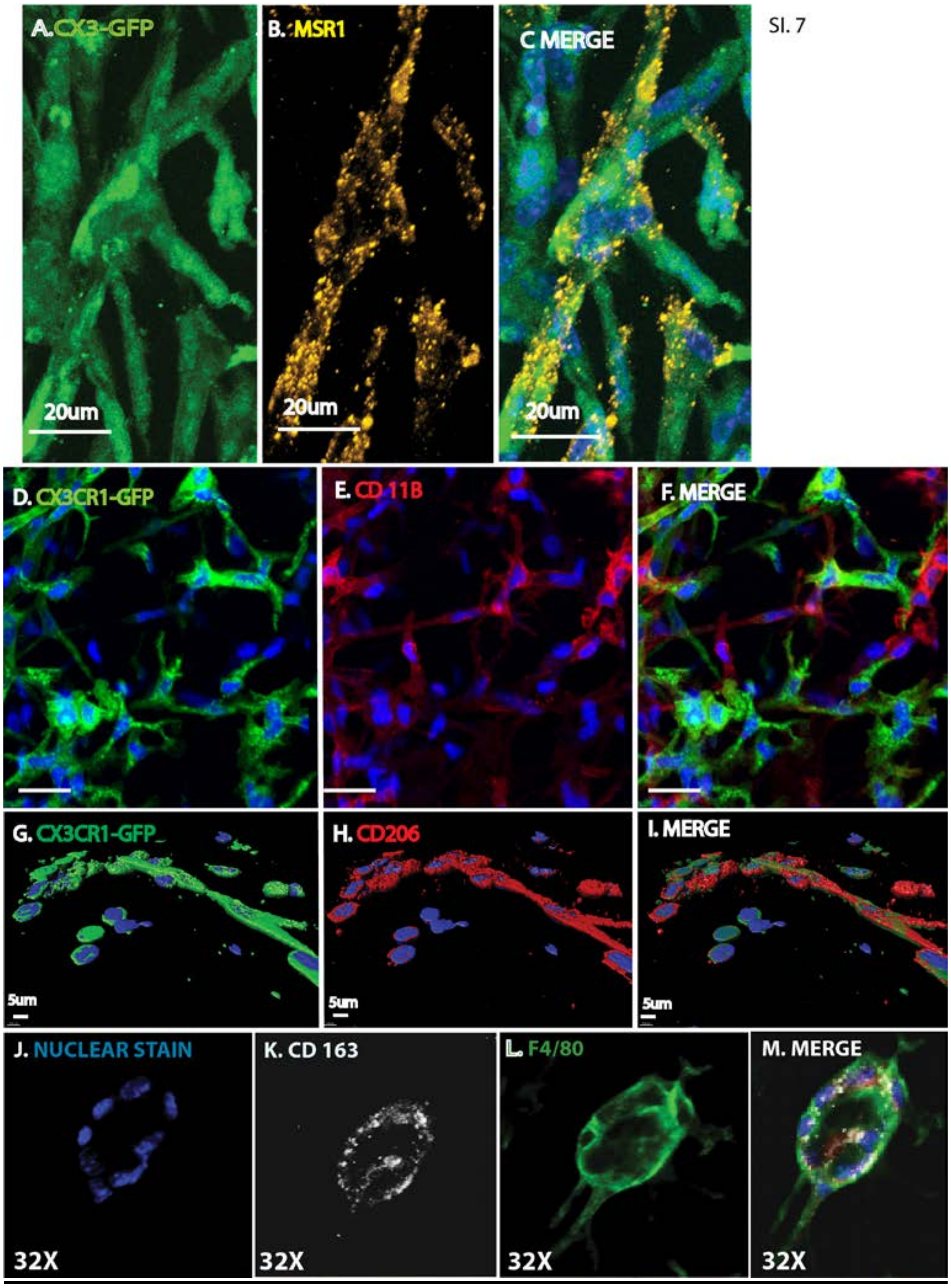

<u>SI.7 CX3CR1$^{GFP/GFP}$ cells express multiple macrophage markers.</u> All images are Z-stacks. (**A-C**) 7 day sc matrigel plug extracted from a CX3CR1$^{GFP/GFP}$ mouse immunostained against MSR1 (macrophage scavenger receptor 1 - gold). (**D-F**) Nine day plug with nuclear stain in blue. (**D**) CX3CR1$^{GFP/GFP}$ cells, (**E**) CD11b (red) and (**F**) merged channels. (**G-I**) Imaris 3D rendering of an LSM Z-stack from a seven day sc matrigel plug. (**G**) CX3CRI-GFP, (**H**) CD206 (mannose receptor – red) and (**I**) merged channels. (**J-M**) Images generated from frozen sections of a sc matrigel plug harvested from a C57BL/6J wild type mouse. The sections were immunostained with antibodies directed against F4/80 (green), CD31 (red), CD163 (white) and counterstained with Hoechst nuclear stain (blue). Scale bars as indicated.

SI. 8

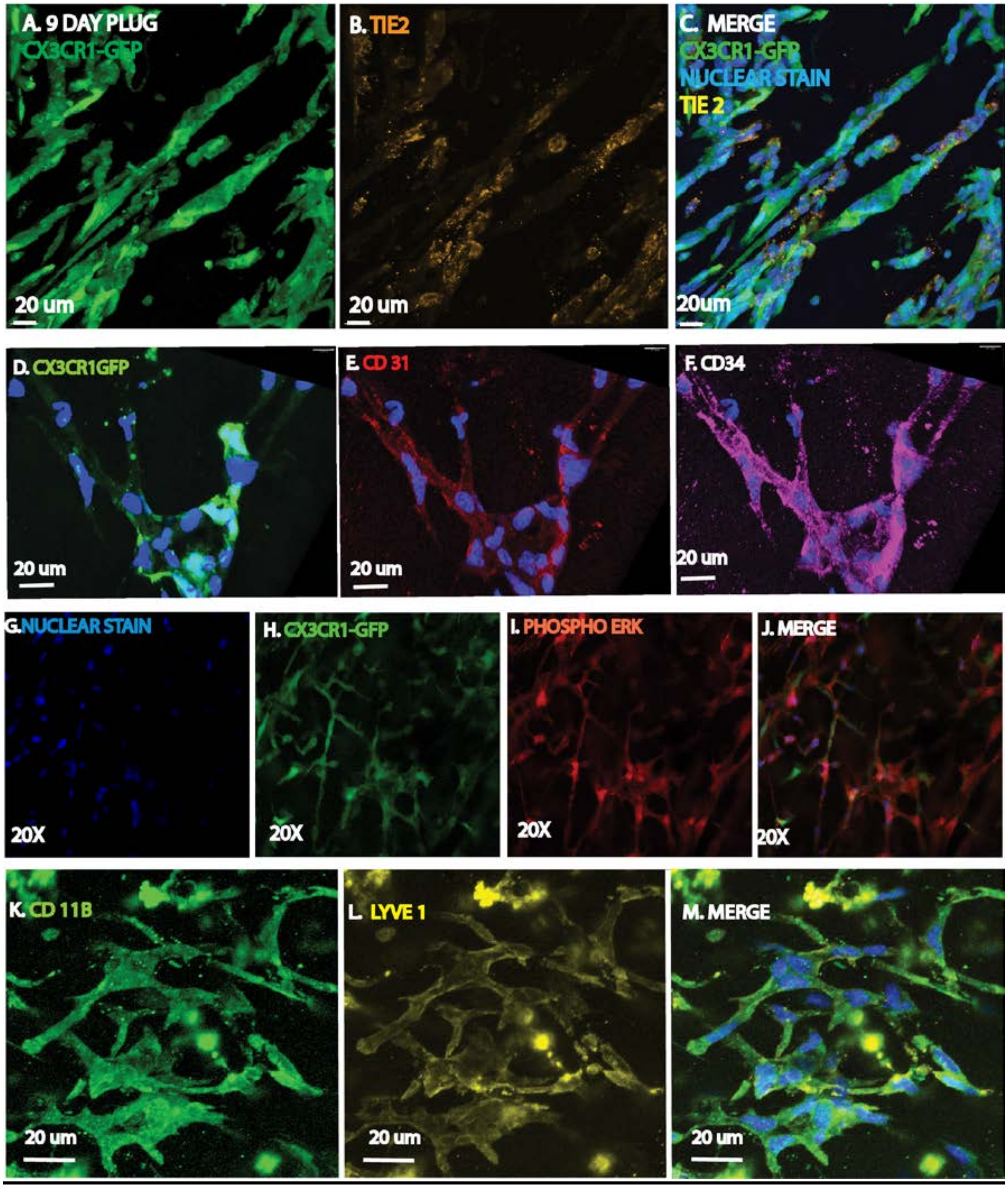

SI 8 Macrophages within the sc plug express Tie2, CD31, CD34, phosphoErk and LYVE1. Images are LSM Z-stacks. Matrigel was sc injected into either CX3CR1$^{GFP/GFP}$ or C57BL/6J mice and plugs explanted 10 days later. (**A-C**) Tie2 expression by CX3CR1$^{GFP/GFP}$ cells. (**A**) CX3CR1$^{GFP/GFP}$ (green), (**B**) Tie2 (gold) and (**C**) merged channels including nuclear stain (blue). (**D-F**) CD31 and CD34 are expressed by CX3CR1$^{GFP/GFP}$cells. (**D**) CX3CR1$^{GFP/GFP}$, (**E**) CD31 and (**F**) CD34. (**G-J**) PhosphoERK is expressed by CX3CR1$^{GFP/GFP}$ cells. (**G**) nuclear stain, (**H**) CX3CR1$^{GFP/GFP}$, (**I**) phosphoERK and (**J**) merged channels. (**K-M**) C57BL/6J 10 day sc plug. (**K**) CD11b, (**L**) LYVE1 and (**M**) merged channels. Scale bars and magnification are shown.

SI. 9

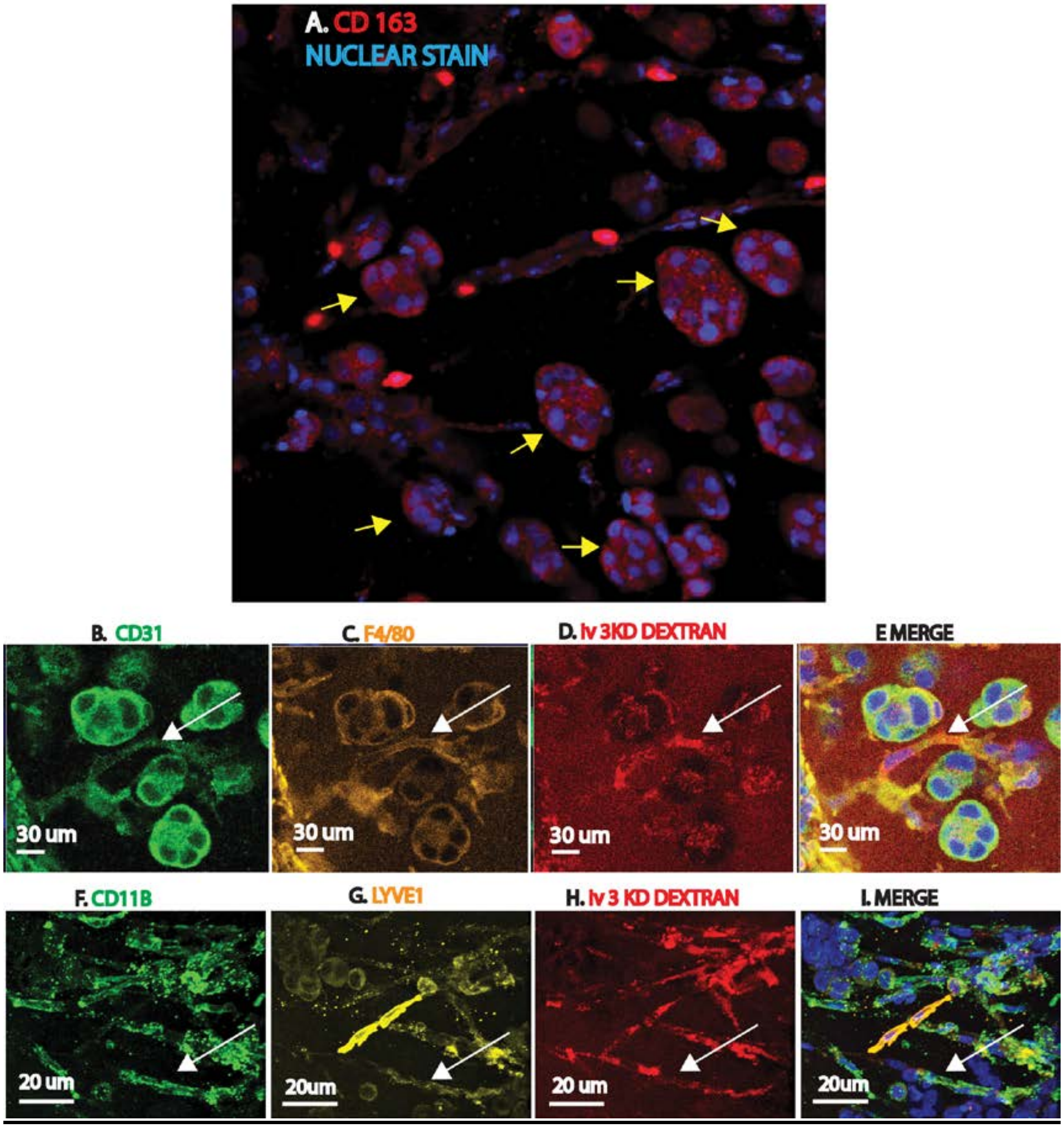

<u>**SI 9 Tumor rosettes are connected to macrophage conduits and are perfused.**</u> Images are LSM Z-stacks (**A-E**) A375 human melanoma tumor cells were injected subcutaneously into an athymic mouse. Seven days later, tumors were explanted and immunostained for various markers. (**A**) Tumor tufts and conduits express CD163 (red) and nuclear stain is in blue. Tufts are identified with yellow arrows. **(B-E)** The mouse was iv injected with 3 KD rhodamine dextran via tail vein, tumor excised and immunostained. (**B**) CD131, (**C**) F4/80, (**D**) intravenous 3 KD dextran and (**E**) merged channels,including the nuclear stain. The white arrow shows a tubular structure that immunostains for CD31, F4/80 and transmits systemically injected dye. (**F-I**) B16/F10 murine melanoma tumor cells were injected sc into a C57BL/6J mouse. Seven days later, tumors were excised and immunostained for **(F)** CD11b, **(G)** LYVE1, **(H)** iv 3 KD rhodamine dextran and **(I)** merged channels with nuclear stain. White arrow shows a tubular structure that stains for CD11b, LYVE1 and transmits iv injected dye. Scale bars as indicated.

SI. 10

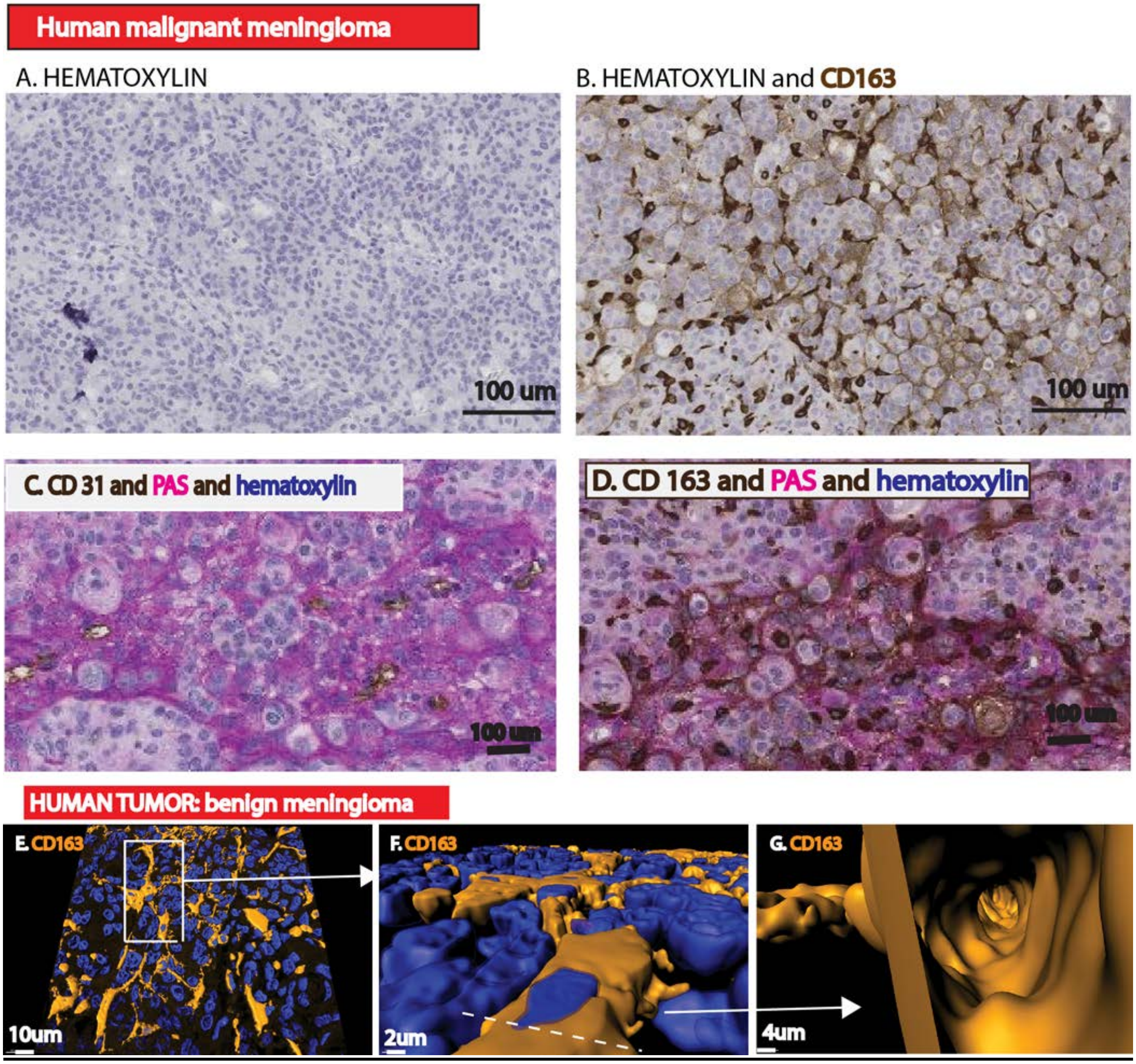

SI.10 Both malignant and benign human meningiomas form an interconnected macrophage network. (A-B) and (C-D) are 6 µm serial sections stained independently from the same region of interest. (**A-B**) A macrophage network seen in (**B**) is present in malignant meningioma. Hematoxylin stain (blue) and CD163 immunoperoxidase (brown). (**C-D**) MALIGNANT meningioma tissue stained with the vascular mimicry marker PAS (magenta) and immunostained with either the endothelial marker CD31 (**C**) or the macrophage marker CD163 (**D**); both shown in brown. Blood vessels, shown in (**C**), are sparse, compared to the robust CD163 network in (**D**). Note colocalization of PAS and CD163. (**E-G**) IMARIS 3D representation of a Z-stack depicting a macrophage network in human BENIGN meningioma. Tissue was

stained with an anti CD163 antibody (gold) and Hoechst, a nuclear stain (blue). (**F**) Perspective view of boxed area in image (**E**). Dashed line represents a transverse cut through this $CD163^{+}$ tubular structure. **(G)** View down the lumen of the same CD163 tubular structure with nuclei removed. Scale bars as indicated.

SI MOVIES

Movie 1 Still image shown in Figure 1I. Movie depicts travel within an F4/80 positive (red), Cx3CR1 $^{GFP/GFP}$ vascular mimicry tube within a sc matrigel plug (NO TUMOR).

Movie 2 Still image shown in Figure 6. A three day subcutaneous A375 tumor was removed from an Athymic mouse after 3 KD rhodamine Dextran (red) was tail vein injected. Tumor tissue was fixed and immunostained for CD11b (purple). Movie depicts travel within the CD11b tube, followed by the addition of the red channel to show that the dye is within the CD11b tube.

Movie 3 Still image is shown in Figure 7. A three day sc A375 tumor was removed from an athymic mouse after tail vein injection of 3KD rhodamine Dextran (red). The tumor tissue was immunostained for CD11b (purple), CD31 (green) and Hoechst nuclear stain (Blue). Movie depicts a confocal Z-stack image composed of the four individual acquired channels. The blue channel is Hoechst, a nuclear stain. The green channel depicts CD31 staining. The red channel shows the tail vein injected dye and finally, the purple channel (far red) depicts CD11b. Note the absence of CD31 staining in some of the perfused CD11b conduits.
